# Mapping genomic regulation of kidney disease and traits through high-resolution and interpretable eQTLs

**DOI:** 10.1038/s41467-023-37691-7

**Published:** 2023-04-19

**Authors:** Seong Kyu Han, Michelle T. McNulty, Christopher J. Benway, Pei Wen, Anya Greenberg, Ana C. Onuchic-Whitford, Nephrotic Syndrome Study Network (NEPTUNE), Dongkeun Jang, Jason Flannick, Noël P. Burtt, Parker C. Wilson, Benjamin D. Humphreys, Xiaoquan Wen, Zhe Han, Dongwon Lee, Matthew G. Sampson

**Affiliations:** 1 Division of Pediatric Nephrology, Boston Children’s Hospital, Boston, MA USA; 2 Department of Pediatrics, Harvard Medical School, Boston, MA USA; 3 Kidney Disease Initiative, Broad Institute, Cambridge, MA USA; 4 Center for Precision Disease Modeling, University of Maryland, School of Medicine, Baltimore, MD USA; 5 Division of Renal Medicine, Brigham and Women’s Hospital, Boston, MA USA; 6 Programs in Metabolism and Medical and Population Genetics, Broad Institute, Cambridge, MA USA; 7 Division of Genetics and Genomics, Boston Children’s Hospital, Boston, MA USA; 8 Department of Pathology and Immunology, Washington University in St. Louis, St. Louis, MO USA; 9 Division of Nephrology, Department of Medicine, Washington University in St. Louis, St. Louis, MO USA; 10 Department of Developmental Biology, Washington University in St. Louis, St. Louis, MO USA; 11 Department of Biostatistics, School of Public Health, University of Michigan, Ann Arbor, MI USA; 12 Manton Center for Orphan Disease Research, Boston Children’s Hospital, Boston, MA USA

**Keywords:** Genome informatics, Gene regulation

## Abstract

Expression quantitative trait locus (eQTL) studies illuminate genomic variants that regulate specific genes and contribute to fine-mapped loci discovered via genome-wide association studies (GWAS). Efforts to maximize their accuracy are ongoing. Using 240 glomerular (GLOM) and 311 tubulointerstitial (TUBE) micro-dissected samples from human kidney biopsies, we discovered 5371 GLOM and 9787 TUBE genes with at least one variant significantly associated with expression (eGene) by incorporating kidney single-nucleus open chromatin data and transcription start site distance as an “integrative prior” for Bayesian statistical fine-mapping. The use of an integrative prior resulted in higher resolution eQTLs illustrated by (1) smaller numbers of variants in credible sets with greater confidence, (2) increased enrichment of partitioned heritability for GWAS of two kidney traits, (3) an increased number of variants colocalized with the GWAS loci, and (4) enrichment of computationally predicted functional regulatory variants. A subset of variants and genes were validated experimentally in vitro and using a *Drosophila* nephrocyte model. More broadly, this study demonstrates that tissue-specific eQTL maps informed by single-nucleus open chromatin data have enhanced utility for diverse downstream analyses.

## Abstract (web summary)

Here, the authors discover over 15,000 eQTLs in human kidney samples by integrating single-nucleus open chromatin data, resulting in high resolution eQTLs, increased enrichment of GWAS heritability and colocalization, followed by downstream validation.

## Introduction

The genomic contributors to kidney diseases and traits extend well beyond rare, pathogenic, exonic variants with large effect sizes that typified the initial discoveries in this area. Focused analyses of individual genes have long illuminated common, non-coding variants whose regulatory effects contribute to their proper function^1,2^. More recently, genome-wide association studies (GWASs) have demonstrated that the heritability of diverse kidney traits and diseases are polygenic and primarily non-coding^3–8^. Thus, whether to deeply understand a single disease-related gene or to fine-map GWAS loci, it is necessary to have as precise an understanding of the genetic control of gene expression as possible. A high-resolution expression quantitative trait loci (eQTL) map of the kidney can contribute greatly to this need, where high-resolution in this context is defined by smaller credible sets and more confidence in prioritized variants.

eQTLs can identify variants associated with gene expression (eSNPs) and their target genes (eGenes) across individuals in a tissue-, and more recently cell-, informed manner^9,10^. In particular, GWAS fine-mapping of diseases and traits has been aided by these eQTL maps given that the non-coding nature of most GWAS variants and linkage disequilibrium (LD) preclude their direct interpretation^11^. Beyond eQTLs, annotations from complementary genomic experiments (e.g., open chromatin peaks) can provide further refinement by identifying functional regions within the disease- and trait-associated loci. Previously, prioritizing GWAS SNPs using eQTL and other functional annotations was achieved by simply overlapping these datasets (*post hoc* lookups). However, investigators have begun to develop new approaches to empower more precise GWAS fine-mapping, particularly by building more high-resolution eQTL datasets^11^.

One method to improve eQTL mapping is to incorporate single-cell data. In a recent study, investigators predicted kidney cell-type interacting eQTLs by applying in silico deconvolution methods using a reference single-cell gene expression dataset and subsequently built a cell fraction-informed eQTL model from bulk tissue^12^. This cell-type-informed eQTL data was then co-analyzed with assay for transposase-accessible chromatin using sequencing (ATAC-seq) data *post hoc* to identify overlaps between specific eSNPs and open chromatin.

Another approach to improve the resolution of eQTL maps is to incorporate functionally-informed SNP annotations in the fine-mapping procedure^13^. As a proof of principle, early studies showed the enrichment of regulatory annotations in eQTLs from lymphoblastoid cell lines. Then, they showed that integrating this information into a Bayesian fine-mapping framework could improve the eQTL discovery and fine-mapping resolution^13,14^. Similarly, a recent study demonstrated increased statistical fine-mapping accuracy of eQTLs by assigning weights to SNPs using priors derived from diverse functional annotations for the subsequent eQTL analysis^15^. This approach increases eQTL discovery and loci for downstream consideration that would have been missed using the *post hoc* lookup strategy.

In this study, we further extend this approach by incorporating single-nucleus open chromatin data from kidney tissue to fine-map kidney eQTLs. Given that SNPs within open chromatin peaks are more likely to impact transcriptional regulation^13^, we hypothesized that weighting SNPs by this parameter would increase eQTL discovery and fine-mapping resolution of putative functional SNPs that otherwise would not be found due to high LD or low allele frequencies. In doing so, we hypothesized that we would gain (1) greater statistical and functional confidence in putative causal eSNPs, (2) additional insight into the regulatory landscape for specific genes related to kidney diseases and traits, and (3) increased discovery and fine-mapping resolution for genome-wide, integrative analyses.

**Fig. 1:**
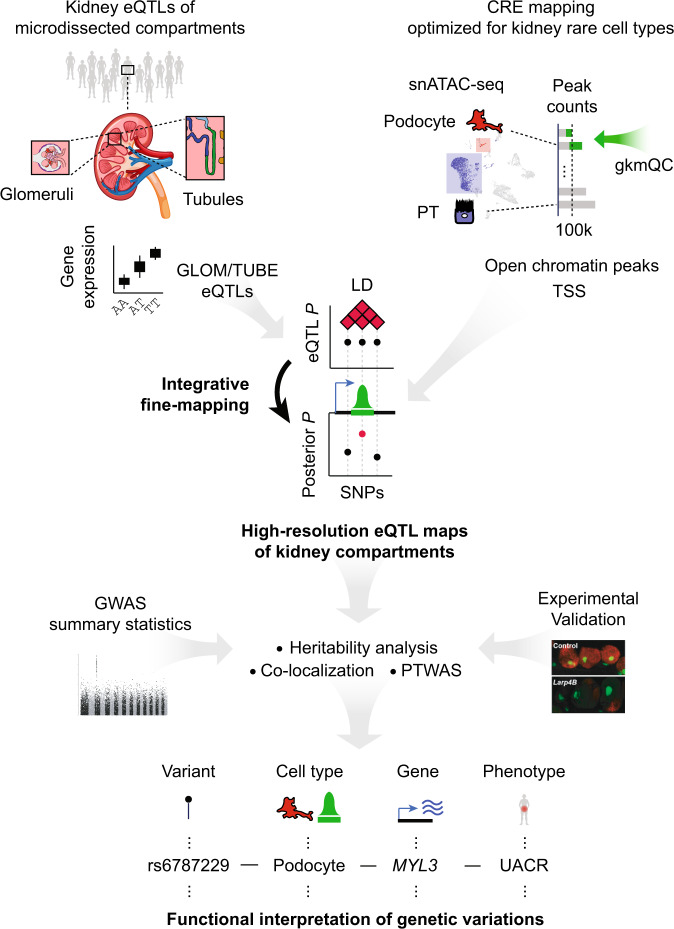
Analysis schematic. Schematic integrating eQTLs with cell-type *cis*-regulatory annotations to build high-resolution eQTL maps of micro-dissected glomeruli (GLOM) and tubulointerstitium (TUBE) and downstream analyses for functional interpretation of genetic variations associated with kidney functional traits. eQTL expression quantitative trait loci, CRE *cis*-regulatory element, snATAC-seq single nuclear assay for transposase-accessible chromatin using sequencing, gkmQC gapped k-mer SVM quality check, TSS transcription start site, LD linkage disequilibrium, SNP single nuclear polymorphism, GWAS genome-wide association study, PTWAS probabilistic transcriptome-wide association study, *MYL3* Myosin Light Chain 3, UACR urine albumin-to-creatinine ratio.

To test this hypothesis, we created a workflow to discover high-resolution eQTLs by using single-nucleus open chromatin data from kidney tissue to generate priors for use in a Bayesian multi-SNP eQTL detection and fine-mapping algorithm (Fig. 1). We applied cell-specific and sequence-based predictive models to these eQTLs to predict regulatory impacts and conducted heritability enrichment analyses, probabilistic colocalization, and transcriptome-wide association studies with GWAS of estimated glomerular filtration rate (eGFR) and urine albumin-to-creatinine ratio (UACR)^6,7^. A subset of candidate eQTLs were validated experimentally in vitro and using a *Drosophila* nephrocyte model. Altogether, we demonstrated improved precision in discerning putative functional SNPs within eSNP haploblocks, which subsequently increased discovery and provided biological insight in downstream analyses. Our interactive resource is available to the public at www.nephqtl2.org.

## Results

### Multi-SNP fine-mapping of *cis*-eQTLs incorporating cell-type open chromatin annotations provides high-resolution eQTL maps

The eQTL analysis consisted of 332 NEPTUNE^16^ individuals with paired RNA-seq and whole-genome sequencing (WGS) data, including 240 glomerular samples (GLOM) and 311 tubulointerstitial samples (TUBE; Supplementary Data 1). For open chromatin annotations, we used kidney cell *cis*-regulatory element (CRE) maps that we recently created through the development and application of a new method to optimize the discovery of rare cell-type specific peaks using underlying sequence signatures - gkmQC^17^.

**Fig. 2:**
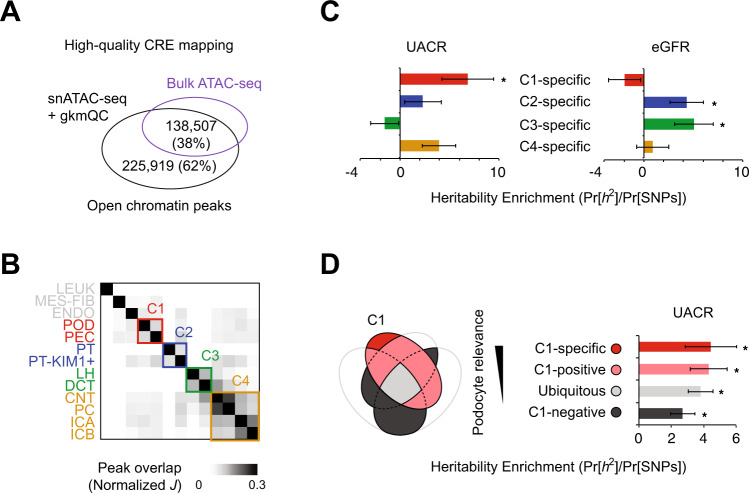
High-quality CREs and their cell-type specific contribution to the heritability of functional kidney traits. **A** Overlap between peaks of kidney snATAC-seq and bulk ATAC-seq from different samples. CRE *cis*-regulatory element, snATAC-seq single nuclear assay for transposase-accessible chromatin using sequencing**. B** Heatmap presents the normalized Jaccard index (*J*) of the peak overlap between cell types used to form cell groups (“Methods”). C1-4 are groups that include kidney cell types from different kidney compartments; C1 = glomerulus, C2 = proximal tubule, C3 = the loop of Henle, C4 = collecting duct. LEUK leukocytes, MES-FIB mesangial/fibroblast, ENDO endothelial, POD podocyte, PEC parietal epithelial, PT proximal tubule, PT-KIM1 + KIM1 positive proximal tubule, LH loop of Henle, DCT distal convoluted tubule, CNT connecting tubule, PC principal cells, ICA type A intercalated cells, ICB type B intercalated cells**. C** The bar graph presents the heritability enrichment partitioned by the genomic coordinates of cell-group-specific open chromatin peaks. Error bars and asterisks depict standard error and significance of the enrichment estimated using block jackknife method. * Stratified LD-score regression (S-LDSC) *P* ≤ 0.05. UACR urine albumin-to-creatinine ratio, eGFR estimated glomerular filtration rate**. D** The bar plot compares the heritability enrichments for urine albumin-to-creatinine ratio for the subgroups of peaks stratified by relevance to the C4 group (POD and PEC). * *P* ≤ 0.05. Exact *P*-values for (C) and (D) are in Supplementary Data 2.

We first compared our optimized single-nucleus kidney CRE maps^17^ to bulk kidney data to show that the single-nucleus data detected 62% additional open chromatin regions not detected in bulk kidney data^18^ (Fig. 2A). Beyond the increased quantity and uniqueness of CREs identified, several metrics indicated that our kidney single-nucleus CRE maps were of high quality. First, the statistical overlap of open chromatin peaks sorted the kidney cell types into four groups (which we denote as “C1” through “C4”) that reflected functional similarities and physical location in the nephron (Fig. 2B). By conducting stratified LD-score regression (S-LDSC)^19^ with cell-group-specific peaks, we found enriched heritability for UACR GWAS variants residing within C1-specific peaks (6.85 fold; *P* = 0.02), which includes peaks specific for podocytes and parietal epithelial cells. eGFR heritability was enriched in SNPs within C2 and C3-specific CREs, which include proximal tubule (*P* = 0.05) and loop of Henle (*P* = 0.04) specific peaks, respectively (Fig. 2C; Supplementary Data 2). Additionally, the heritability enrichment for UACR increased with respect to groupings of peaks with higher podocyte and parietal epithelial cell specificity (Fig. 2D).

**Fig. 3:**
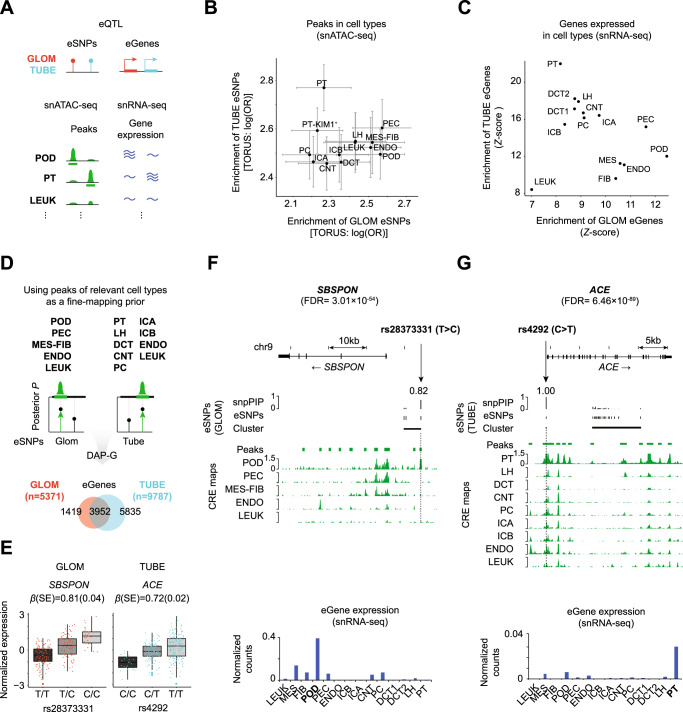
Integrative analysis of eQTLs and high-quality CRE maps. **A** Schematic demonstrates the cell-type-specific enrichment of eSNPs and eGenes for the peaks and genes from snATAC/RNA-seq datasets. eQTL expression quantitative trait loci, eSNPs single nucleotide polymorphisms associated with gene expression, eGenes genes with at least one variant associated with expression, GLOM/TUBE glomerular/tubulointerstitial eQTLs, snATAC-seq single nuclear assay for transposase-accessible chromatin using sequencing, snRNA-seq single nuclear RNA sequencing. **B** Enrichment analyses of eSNPs in open chromatin peaks in corresponding cell types from matched sample eQTL analysis (N_GLOM_ = N_TUBE_ = 219). Horizontal (GLOM) and vertical (TUBE) bars represent 95% confidence interval for the enrichment estimate. log(OR) natural logarithm of the odds ratio estimated by TORUS. **C** Enrichment analysis of eGenes among all genes expressed in corresponding cell types. **D** Cell types used for CRE fine-mapping annotations for integration into deterministic approximation of posteriors (DAP-G) and resulting eGenes. snpPIP SNP posterior inclusion probability**. E** Plots of the top ranked GLOM (*N* = 240) and TUBE (*N* = 311) specific eQTLs. The box plots contain the 25th−75th quartile with median indicated by the middle bar. Lines extend 1.5 times the interquartile range. *SBSPON* Somatomedin-B and thrombospondin type-1 domain-containing, *ACE* Angiotensin-converting enzyme, β (SE) Effect size of genotype on gene expression and standard error from single-SNP association. **F**,**G** Specific examples of fine-mapped eSNPs (hg19 coordinates) in the clusters of top GLOM/TUBE-specific eGenes. The heights of vertical black bars depict the snpPIP of each clustered eSNP. Horizontal black bars depict the genomic range of each cluster. Green horizontal bars depict the range of open chromatin peaks of the relevant cell types. The green vertical graph shows the normalized pile-up of snATAC-seq reads of the corresponding cell type. Blue bar plots present the gene expression of snRNA-seq data normalized by genes and cell counts. POD podocyte, PEC parietal epithelial, MES-FIB mesangial+fibroblast, ENDO endothelial, LEUK leukocytes, PT proximal tubule, LH loop of Henle, DCT/CNT distal convoluted / connecting tubule, PC principal cells, ICA/ICB type A/B intercalated cells, PT-KIM1 + KIM1 positive proximal tubule.

We used MatrixEQTL^20^ for our single-SNP eQTL analysis, a necessary precursor to the ultimate multi-SNP fine-mapping step. The effect sizes from these results were comparable to those from eQTL studies of bulk kidney cortex and of GLOM and TUBE from nephrotic syndrome (NS)^21,22^ and non-NS^23^ samples (Supplementary Fig. 1). A principal component analysis of eQTL *z*-scores from GLOM, TUBE, and GTEx tissues found that GLOM and TUBE were proximal to the kidney cortex and other non-brain tissues (Supplementary Fig. 2A). GLOM eSNPs showed the highest enrichment in podocyte and parietal epithelial cell open chromatin peaks, and TUBE eSNPs showed the highest enrichment in proximal tubule peaks (Fig. 3A, B). Cell-type enrichment is also higher than randomly expected (Supplementary Fig. 2B). Of note, SNPs identified uniquely by our optimized peak calling method significantly contributed to cell-type enrichment (Supplementary Data 3). Similarly, GLOM eGenes were most enriched for expression in podocyte and parietal epithelial cells, and the TUBE eGenes were most enriched for expression in proximal tubule cells (Fig. 3C). Finally, in contrast to kidney cell types, no tissues from GTEx or ENCODE had a significant enrichment bias towards either GLOM or TUBE eSNPs^24,25^ (Supplementary Fig. 2C, D). This indicates that the kidney single-nucleus-based approach is necessary to create functional annotation priors discriminating transcriptional landscapes of GLOM and TUBE eQTLs.

We generated SNP priors by including the union of our cell-type-specific open chromatin annotations (pseudo-bulk) and the enrichment based on the distance between each SNP and the corresponding gene’s transcription start site (TSS). We refer to this SNP Prior as the “integrative prior” (see Methods). For our GLOM CRE annotation, all SNPs within podocyte, parietal epithelial, endothelial, mesangial/fibroblast and leukocyte open chromatin peaks were combined. For TUBE, we combined all SNPs within CRE from all proximal tubule clusters, loop of Henle, distal convoluted tubule, connecting tubule, principal cells, type A and B intercalated cells, in addition to the same endothelial and leukocytes annotations used for GLOM (Fig. 3D). We determined the weights for each SNP by integrating the estimated enrichment of our single-SNP eQTL associations among CREs with TORUS^26^. Variants in open chromatin are 3.95 [CI: 3.76, 4.15] fold more likely to be eSNPs in the TUBE and 4.03 [CI: 3.72, 4.35] fold more likely in the GLOM.

The integrative priors were then used with our expression and WGS data to fine-map*cis*-eQTLs allowing for multiple independent SNPs per gene using the method DAP-G^27,28^. After Bayesian FDR control, we identified 5,371 GLOM and 9,787 TUBE eGenes (Fig. 3D, Supplementary Data 4). This is an approximately 6-fold increase in eGenes compared to our previous array-based analysis^21^, and about 2.5-fold when subset to overlapping samples, demonstrating that the increase in discovery is not solely from the increased sample size (Supplementary Fig. 3). Of note, the increased dynamic range of RNA-seq (compared to array) and genotype calling accuracy with higher-depth WGS may also have contributed to the increase in eGenes. When comparing our eGenes to other public datasets, 76.5% and 69.3% of our TUBE and 52.2% and 52.1% of our GLOM eGenes were replicated from Qiu et al.^23^, and GTEx kidney cortex^22^, respectively. Demonstrating replication of our fine-mapping methods, we found increased correlation of eSNP ranking (from independent subsets, see Methods) using our fine-mapping technique compared to ranking of SNPs from the single-SNP eQTL analysis with MatrixEQTL (Supplementary Fig. 4).

Our increase in resolution and interpretability is illustrated with the Somatomedin B And Thrombospondin Type 1 Domain Containing gene (*SBSPON*), the most significant GLOM-specific eGene (FDR = 3.01 × 10^−54^; Fig. 3E, F). Before fine-mapping, we found three eSNPs within the single associated haploblock with indistinguishable effects on *SBSPON* expression (Supplementary Data 5). However, our multi-SNP fine-mapping identified rs28373331 as the putative causal eSNP (SNP posterior inclusion probability (snpPIP) = 0.82). Inspection of the snATAC-seq data identified that this SNP was in a podocyte-unique open chromatin peak ~10 kb upstream of the *SBSPON* locus. This is concordant with the snRNA-seq analysis showing its podocyte-specific expression (Fig. 3F).

Angiotensin I Converting Enzyme (*ACE*) is a TUBE-specific eGene with one of the top-ranked signals (FDR = 6.46 × 10^−89^; Fig. 3E, G). Our fine-mapping also found a putatively causal eSNP (rs4292; snpPIP = 1.00) in the *ACE* promoter that is open across multiple cell types, with the strongest peak in proximal tubules, which is concordant with its gene expression pattern. Taken together, our results suggest that this cell-type CRE-informed fine-mapping approach provides higher resolution eQTL maps, which improves our ability to dissect the transcriptional regulation in kidney tissues.

### CRE-informed eQTLs provide higher-resolution fine-mapping and enriched heritability for kidney phenotypes

We next conducted a series of complementary genome-wide analyses to assess potential improvements in eQTL fine-mapping resolution that result from using an integrative prior versus a (1) uniform prior—all SNPs having equal prior probability of being an eSNP, and (2) TSS prior—only including the distance from the TSS.

**Fig. 4:**
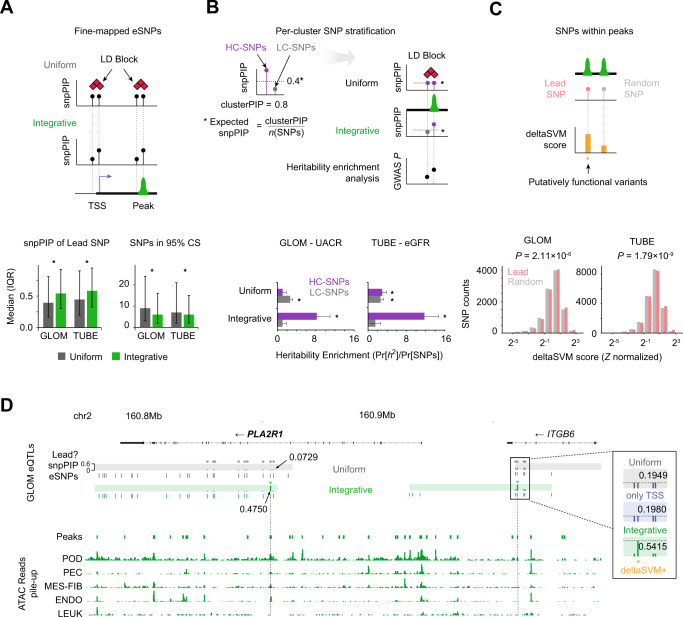
Specifying putative functional variants using the high-resolution eQTL map. **A** A schematic showing how fine mapping with an integrative prior stratifies putative functional variants among eSNPs within the same linkage disequilibrium (LD) block (top). For each eGene cluster in GLOM and TUBE, we compare the distribution of the top SNPs’ snpPIPs and the number of SNPs forming the 95% credible set between the uniform and integrative priors (bottom). The bar height and whiskers indicate the median and interquartile range (IQR). The distributions are significantly different for all four comparisons, two-sided, unpaired Wilcoxon rank sum test *P* < 2×10^−16^. The number of credible sets used for each analysis are: N_GLOM-Uniform_ = 3572, N_GLOM-Integrative_ = 3788, N_TUBE-Uniform_ = 7757, N_TUBE-Integrative_ = 8775. eSNPs single nucleotide polymorphisms of eQTL, snpPIP SNP posterior inclusion probability, TSS transcription start site, CS credible set. **B** Schematic demonstrates how high/low-confidence eSNPs (HC/LC-SNPs) are stratified per cluster and how the integrative prior weighs the putative functional SNPs enriched for the heritability of relevant traits (top). Bar plots compare the heritability enrichment of GLOM/TUBE HC/LC-SNPs fine mapped with uniform or integrative priors (bottom). Asterisks depict the significant enrichment of the heritability assessed by stratified LD score regression (S-LDSC) using block jackknife method, *P* ≤ 0.05 (Supplementary Data 6). UACR urine albumin-to-creatinine ratio, eGFR estimated glomerular filtration rate. **C** Diagram demonstrating hypothesis that high-resolution eQTLs are more likely to be functional than matched random common SNPs within open chromatin peaks. Difference between deltaSVM scores is assessed by the two-sided Wilcoxon rank-sum test. **D** Genomic coordinate diagram comparing eSNP fine-mapping with the integrative or uniform priors at Phospholipase A2 Receptor 1 (*PLA2R1*). In each cluster, using the integrative prior (green) resolves fine-mapping ambiguity and identifies a putative functional eSNP in open chromatin. Asterisks mark the lead SNP per cluster and arrows indicate snpPIP scores. In right cluster, using the integrative prior identifies one deltaSVM positive (orange) lead eSNP compared to snpPIPs from the uniform (gray) and TSS-only priors (blue). Horizontal and vertical bar plots depict the genomic ranges of open chromatin peaks and the pile-up of snATAC-seq reads for cell types: *ITGB6* Integrin Subunit Beta 1.

The first metric was the posterior probability of each cluster’s lead SNP (snpPIP), where an increase would indicate more confidence being shifted to the lead SNPs. The second metric was the number of SNPs in the eQTL’s 95% credible sets, where a decrease would indicate an improvement. On both metrics, the integrative prior showed the highest fine-mapping resolution, followed by the TSS prior alone, then a uniform prior (Fig. 4A; Supplementary Fig. 5A).

The third metric was a change in S-LDSC-based heritability enrichment of high confidence (HC) versus low confidence (LC) eSNPs as a function of prior choice. We defined HC-SNPs as those whose posterior inclusion probability (PIP) within a haploblock cluster is higher than would be observed if the PIPs were equally distributed among all SNPs in a locus. LC-eSNPs have snpPIPs smaller than expected (Fig. 4B). There was a significant enrichment of heritability for HC-SNPs using the integrative prior for UACR and eGFR; this was greater than the heritability observed in analyses using the uniform prior. The HC-SNPs from the integrative prior had more enriched heritability compared to LC-SNPs in primary kidney phenotypes^7,29,30^ and negative control phenotypes^31^ (Supplementary Fig. 5B, Supplementary Data 6). Notably, GLOM HC-SNPs had significantly-enriched heritability for high-powered GWAS phenotypes related to immune cell counts, potentially attributed by cell-type-agnostic regulatory variants (discussed below).

The final metric was a change in the computationally-predicted functionality of fine-mapped eSNPs defined using the different priors. To do this, we compared the deltaSVM score of lead eSNPs to random SNPs in the CREs, controlling for allelic frequency, distance from TSS, and the signal strength of open chromatin peaks. Lead eSNPs from the integrative prior had significantly higher deltaSVM scores than the random SNPs (*P* = 2.22 × 10^−9^ for GLOM; *P* = 4.64 × 10^−6^ for TUBE; Wilcoxon rank-sum test; Fig. 4C) and those fine-mapped by uniform priors (*P* = 5.65 × 10^−7^ for GLOM; *P* = 1.49 × 10^−17^ for TUBE; Supplementary Fig. 5C).

A focused analysis of *PLA2R1* illustrates the power of these high-resolution eQTL maps to identify multiple independent associations and more accurately specify the putative functional variants. *PLA2R1* is a glomerular gene specifically expressed in podocytes (Supplementary Fig. 5D) that is associated with a rare kidney disease, membranous glomerulonephritis^32^. Our fine-mapping identified four independent eSNP clusters in GLOM. We highlight two clusters in Fig. 4D where the lead eSNPs identified using the integrative prior have a higher posterior probability than those identified using the uniform prior. The lead SNPs from the integrative prior were in podocyte-specific open chromatin peaks, concordant with the gene expression pattern of *PLA2R1*, and are deltaSVM positive, implying that the lead SNPs newly found using the integrative prior are more likely to be causal variants.

### Colocalization of high-resolution eQTLs and kidney-relevant GWAS SNPs identifies novel genes and increased resolution of colocalized signals

Including cell-type-informed CREs as integrative priors in our eQTL analyses led to increased posterior SNP probabilities of putative regulatory SNPs and, in some cases, distinguished between SNPs in high LD. Given this, we hypothesized that colocalization analysis with well-powered GWAS of eGFR and UACR and these high-resolution eQTL maps would increase detection and fine-mapping resolution of colocalized signals. To identify colocalized SNPs, we used fast enrichment estimation aided colocalization analysis (fastENLOC)^33,34^. For eGFR, we identified 46 TUBE and 6 GLOM colocalization signals, which we defined as having a regional colocalization probability (RCP) ≥ 0.5. For UACR, there were 9 TUBE and 21 GLOM colocalization signals (Supplementary Data 7, Fig. 5A**;** Supplementary Fig. 6A-B). Our multi-SNP fine-mapping method also enabled us to identify genes with multiple independent colocalized signals (Supplementary Fig. 7). When comparing colocalization signals for kidney traits derived from previous array-based kidney eQTLs^6,7^, we replicated five genes for UACR – *PRKCI*, *TGFB1*, *PTH1R*, *MUC1*, *OAF* – and 3 for eGFR – *FGF5*, *MLLT3*, *UMOD*. Using the high-resolution eQTLs, we discovered 82 colocalized loci, with 22 of them fine mapped to a single variant. 90% of these single variants (18/22) were in open chromatin. In contrast, we only identified 69 colocalized loci with the uniform prior. Finally, from a systematic comparison of extended sets of colocalized SNPs (RCP ≥ 0.2) across the different priors, we confirmed a significant increase in colocalization probability when high-resolution eQTLs are incorporated (Supplementary Fig. 8).

**Fig. 5:**
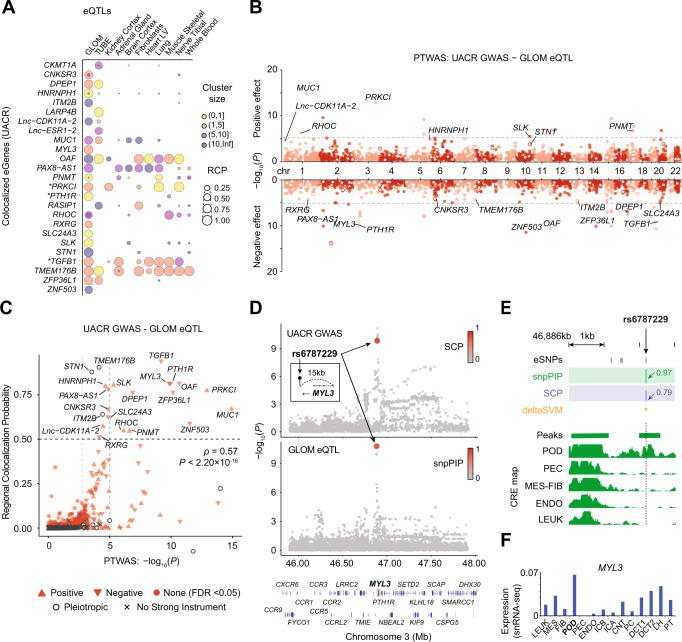
PTWAS and colocalization analyses of GLOM high-resolution eQTLs and UACR GWAS. **A** Bubble plot shows GLOM or TUBE eGenes colocalized with UACR GWAS loci (≥1 cluster with regional colocalization probability (RCP) ≥ 0.5) along with GTEx results from selected tissues, including kidney cortex. Each circle represents an eQTL cluster, and diameter and color depict the RCP scores and the cluster size (number of eSNPs), respectively. * gene replicated from previous analyses, all other genes are novel. **B** A Miami plot shows the PTWAS signal of putative-causal genes associated with the variance of UACR measured by the eQTL-imputed transcriptional changes. Genes lacking a strong instrument are excluded. Each dot represents the gene, an open circle indicates the genes potentially confounded by pleiotropic effects. -log_10_(P)-values represent the result of a generalized burden test for each gene. Dashed lines indicate thresholds using two multiple-testing correction methods (*q*-value ≤ 0.05; light gray) and Bonferroni (*P* ≤ 9.52 × 10^−6^; dark gray). GLOM eGenes in (A) are annotated. **C** A scatter plot of RCP of top colocalized clusters from fastENLOC and corresponding PTWAS associations for each eGene. Point shape depicts the type of PTWAS association’s effect. PTWAS -log_10_(P)-values represent the result of a generalized burden test for each gene. The significance (P) of the correlation coefficient (ρ) between PTWAS and RCP was computed by Pearson-R test. **D** Summary plots of GWAS and eQTL show SNP associations from the UACR GWAS and GLOM eSNPs in the *cis*-window (≤1 Mb from *MYL3* gene body). GWAS hits and eSNPs are colored by SNP-level colocalization probability (SCP) from fastENLOC and snpPIP from eQTL fine-mapping, respectively. GWAS and eQTL -log_10_(P)-values represent single-SNP association tests based on linear regression models. **E** Top: Four eSNPs in the top fastENLOC cluster of *MYL3*. Green and purple vertical bar plots depict the snpPIP and SCP, respectively. Orange asterisk depicts the putative-functional variants inferred by the deltaSVM. Bottom: Green horizontal and vertical bar plots depict the genomic range of open-chromatin peaks and the pile up of snATAC-seq reads on the cell types. **F** Blue bar plots present the normalized expression of *MYL3* in snRNA-seq data.

Previous studies have shown that functional GWAS variants are enriched in CREs of relevant tissues and cell types^35^. Thus, we hypothesized that if a specific prior better captured the functional GWAS variants, then lead colocalized SNPs weighted by that prior would be enriched in CREs more than expected. Indeed, we found that colocalized SNPs found using our high-resolution eQTLs were more enriched for SNPs in open chromatin when compared to the uniform prior. This enrichment was most significant when colocalizing TUBE eQTLs with primary kidney phenotypes^7,29,30^ (Supplementary Fig. 9, Supplementary Data 8). Our results suggest that the CRE-informed colocalization analysis promotes the discovery of the functional GWAS variants. Colocalization results for various publicly available kidney-phenotype GWAS can be found in Supplementary Data 6.

These high-confidence potential target genes and SNPs discovered by our colocalization analyses can be a starting point for mapping GWAS variants to their function. For example, the replication of *PTH1R* highlights the increased resolution and cell-type interpretation. Compared to Teumer et al. we refined the *PTH1R* eSNP credible set size from 14 to 1. The lead SNP also changed from rs73065147 (SNP colocalization probability (SCP) = 0.2), which does not fall within any CRE, to rs6787229 (SCP = 0.79), which resides in a podocyte-specific peak (Supplementary Fig. 10). Thus, by weighing eSNPs within CREs, we more confidently identified putative causal SNPs and hypothesized an association between podocyte-specific regulation of *PTH1R* and UACR.

### Probabilistic transcriptome-wide association analysis (PTWAS) with high-resolution eQTLs identifies associations between SNP-predicted gene expression and kidney phenotypes

Using our high-resolution eQTL maps for the predictive model of gene expression, we next analyzed the association between SNP-predicted gene expression and eGFR and UACR using probabilistic transcriptome-wide association study (PTWAS)^36^ (see Methods). For eGFR, at a false discovery rate (FDR) ≤ 5%, we identified 601 significant gene-trait pairs in GLOM and 1,074 in TUBE. For UACR at an FDR ≤ 5%, we identified 137 significant gene-trait pairs in GLOM (Fig. 5B) and 179 in TUBE (Supplementary Data 9, Supplementary Fig. 11A–C). We also found a significant correlation ( *ρ* = 0.57, *P* ≤ 2.2 × 10^−16^) between colocalization and PTWAS signals (Fig. 5C, Supplementary Fig. 11D–F), demonstrating the consistency of inference results when different analytical approaches are applied to the same dataset^37^.

Our integrative analysis enables us to interpret cell types and CREs in which GWAS variants regulate their target genes. As a representative case, we highlight a colocalized SNP, rs6787229, associated with *MYL3* gene expression and UACR also validated by PTWAS (*P* = 1.44 × 10^−10^, SCP = 0.79; Fig. 5D). A podocyte-specific open chromatin peak harboring this SNP increased probability of the eQTL fine-mapping (snpPIP = 0.97) compared to fine-mapping with the uniform prior (snpPIP = 0.81) (Fig. 5E). This inferred cell-type specificity was corroborated by podocyte-specific gene expression of target gene *MYL3* (Fig. 5F). Taken together, these integrative approaches with high-resolution eQTL maps increase the opportunity to map variants to function via gene regulation with greater interpretability and confidence.

### SNP- and gene-level validation of predicted-causal eQTLs results in reduced *Drosophila* nephrocyte function and SNP-level regulation of *LARP4B* and *NCOA7*

**Fig. 6:**
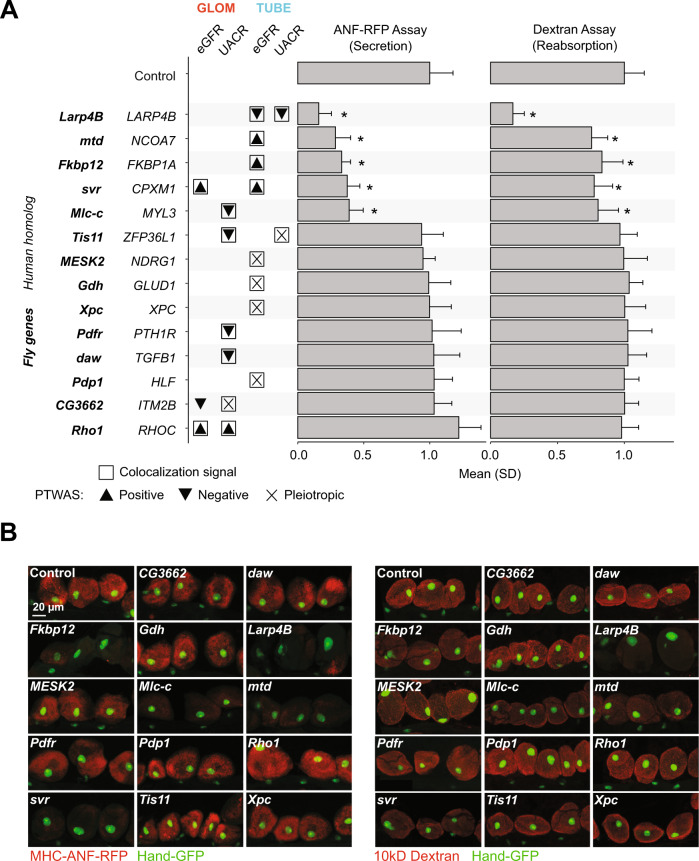
The impact on *Drosophila* nephrocyte function caused by RNAi of the kidney trait-associated genes. **A** Comparison of 14 genes and RNAi control. *n* = 60 nephrocytes from 3 biologically independent *Drosophila* (20 nephrocytes per *Drosophila*). *Drosophila* genes are followed by human homologs and association indicator from tissue-phenotype analyses: colocalization (RCP ≥ 0.5) and PTWAS associations (FDR ≤ 0.05), including direction of effect. Bar plots indicate mean and standard deviation (SD) of fluorescence of nephrocytes. Significant differences (two-sided t-test) between the gene and control are indicated (*P ≤ 0.001). Exact *P* values are in Supplementary Data 10. **B** Fluorescence microscopy images for representative nephrocytes for each *Drosophila* gene. Left panel: MHC-ANF-RFP assay. Right panel: 10kD Dextran; Hand-GFP nephrocyte nucleus. The scale bar is 20 µm, and it is the same scale for all the panels in this figure.

We identified GLOM and TUBE eGenes that were (1) significant in both colocalization and PTWAS analyses with UACR and/or eGFR, (2) contain colocalized SNPs in CREs, and (3) had gene homolog expression in *Drosophila* nephrocytes. Fourteen of these 32 resulting genes were randomly selected for experimental validation (Supplementary Data 10;  see Methods). To do this, we used an in vivo *Drosophila* model. The *Drosophila* nephrocytes filter and reabsorb circulating proteins in the hemolymph and share many similarities with glomeruli and tubule cells at the functional, molecular, and ultrastructural levels^38,39^, making it an ideal model for both GLOM and TUBE eGenes. In flies carrying MHC-ANF-RFP transgene, the myosin heavy chain (MHC) promoter directs muscle cell expression of a rat atrium natriuretic factor (ANF)–red fluorescent protein (RFP) fusion protein (ANF-RFP) that is secreted into the hemolymph^40^. ANF-RFP is typically filtered and endocytosed by healthy, wild-type nephrocytes, and the intracellular red fluorescence can be readily visualized and quantitated in vivo. We found nephrocyte-specific knockdown of five genes impacted nephrocyte function—*Fkbp12* (*FKBP1A*), *Larp4B* (*LARP4B*), *Mlc-c* (*MYL3*), *mtd* (*NCOA7*), and *svr* (*CPXM1*) (Fig. 6). In an independent ex vivo functional assay, we tested the ability of dissected nephrocytes to absorb Texas Red-labeled 10 kD Dextran particles. Consistent with the secreted protein reabsorption assay, silencing anyone of the five genes resulted in a decrease in intracellular Texas Red fluorescence compared to control nephrocytes. Of note, six genes tested in this experiment (*ZFP36L1*, *NDRG1*, *GLUD1*, *XPC*, *HLF*, and *CG3662*) were assessed as pleiotropic in the PTWAS and were not found to impact nephrocyte function when knocked down. To further validate TUBE associations identified in *LARP4B* and *NCOA7*, we generated luciferase reporter constructs in both forward and reverse orientations (Supplementary Data 11) to test the allele-specific enhancer activity of the lead variants associated with each gene, rs80282103 and rs11154336, respectively. Consistent with *LARP4B* eQTL findings, the rs80282103-T minor allele demonstrated 126% increased reporter activity in HK-2 cells, a human proximal tubule cell line (*P* = 5.99 × 10−^9^). The rs11154336-A allele demonstrated 198% increased reporter activity compared to the G allele, consistent with *NCOA7* eQTL results (*P* = 1.30 × 10−^16^) (Supplementary Fig. 12).

## Discussion

In mapping the non-Mendelian genomic basis of kidney traits and diseases, we are challenged to maximize the detection of regulatory circuits - functional genetic variants in *cis*-regulatory elements, their target genes, and their cells of action. Conventional fine-mapping approaches, depending on the population size and haplotype structure, may be suboptimal in specifying the putative functional variants with low allelic frequency or multiple indistinguishable tag SNPs within the same haplotype block. Given that the functional characteristics of variants in CREs are orthogonal to LD patterns, diverse functional annotations have been used for fine-mapping of GWAS and eQTL variants. To this point, we created a workflow that used single-nucleus open chromatin data to generate priors for use in Bayesian multi-SNP eQTL detection algorithms. In doing so, we demonstrated improved precision in discerning putative functional SNPs within eSNP haploblocks (fine-mapping), which subsequently increased discovery and biologic insight of downstream analyses.

The CRE-informed fine-mapping for eQTLs can recover underpowered variants with a Bayesian approach that augments the enrichment of eQTLs across tissue-relevant cell-type CREs, reflecting the underlying biology of transcriptional regulation. Corroborating this, we found that the gain in heritability and deltaSVM score enrichment, when comparing our integrative approach to results from a uniform prior, was larger in the lower-powered tissue, GLOM, compared to our higher-powered TUBE analysis (Fig. 4). In sum, these integrative approaches can complement the mapping of eQTLs with moderate statistical power and be an effective resource for the discovery of eQTLs from the limited samples with nephrotic syndrome.

A particularly important aspect of this study was our ability to use our newly developed method, gkmQC, to characterize CREs in rare cell types at a coverage and level of resolution not previously attained. For example, it was critical to comprehensively map (putatively)-casual regulatory variants of podocytes, a key rare (<1%) cell type involved in kidney filtration function. By nearly doubling the podocyte-specific peaks, we increased the statistical power of enrichment between podocyte peaks and glomerular eQTLs, which in turn improved fine-mapping efforts. This directly led to our ability to discover, via LD score regression analysis, a significant contribution of podocyte regulatory elements to the heritability of urine albumin-to-creatinine ratio (UACR) from GWAS studies. Interestingly, we also found enrichment of eGFR-associated SNPs among proximal tubule, loop of Henle, and distal convoluted tubule open chromatin. Together, these underscore the ability of our optimized CRE maps to provide biological insights into multiple kidney phenotypes and should serve as a resource to investigators seeking to discern the specific regulatory circuits of these diseases and traits.

As intended, our integrative approach allowed us to increase fine-mapping resolution, marked by smaller credible set sizes and increased statistical confidence of lead SNPs. To highlight the utility of our eQTLs in mapping variants to their function, we performed colocalization and transcriptome-wide association studies with functional kidney outcomes UACR and eGFR. Colocalization analyses are hindered by low power, especially when LD matrices are not perfectly matched^41^. We found that weighing putative functional SNPs in our eQTL analysis resulted in an enrichment of colocalized SNPs within CREs and increased discovery of novel loci, thus partially overcoming this power limitation. These complementary analyses not only highlight the utility of our eQTL resource but also allow for new biological insights into associations between tissue and cell-specific gene regulation and kidney function. This was illustrated by a high confidence eSNP within a podocyte-specific CRE that was associated with both *MYL3* glomerular expression and UACR.

We demonstrated CRE-informed fine-mapped GLOM/TUBE eQTLs had an enriched heritability and colocalization for GWAS traits with cell-type relevance (Figs. 4, 5). However, it is unclear how the statistical power of eQTL/GWAS studies affects heritability and colocalization analyses. We found that eSNPs from the lower-powered GLOM eQTL analysis had high heritability and colocalization enrichment for several negative-control traits from high-powered GWASs (Supplementary Fig. 5B; Supplementary Fig. 9). Those results lead us to hypothesize that high-powered GWAS studies might increase heritability enrichment from tissue-agnostic regulatory SNPs. High-powered GWAS can identify more significant SNPs at lower allele frequencies^42–44^ which can have a pleiotropic, tissue-agnostic impact on gene expression^45^. In contrast, high-powered eQTL studies can identify more tissue-specific regulatory SNPs^46^. Thus, an eQTL analysis with increased GLOM samples may identify more tissue-specific eQTL that robustly discriminate heritability/colocalization signals from UACR/eGFR and the negative-control traits. Given that statistical power differentially impacts GWAS and eQTL studies^47^, differences in statistical power should be considered when interpreting the cell-type relevance of a trait using colocalization/heritability analyses.

By following up on statistical findings in the *Drosophila* nephrocyte, we were able to further validate selected genes of interest. For example, we replicated the association between a single intronic *LARP4B* eSNP, rs80282103, and both UACR and eGFR, previously discovered by Wuttke et al. and Morris et al.^7,48^ and identified a novel association between an intronic *NCOA7* eSNP rs11154336 and eGFR. We found that knockdown of *Larp4b* and *Mtd*, an *NCOA7* ortholog, in the *Drosophila* nephrocyte had the most statistically significant reductions in nephrocyte function, providing orthogonal support for the functional role of *LARP4B* and *NCOA7* in the kidney. Using a luciferase assay, we also validated the functional impact of rs80282103 and rs11154336. In addition to *LARP4B* and *NCOA7*, three novel colocalized genes —*FKBP1A*, *CPXM1*, and *MYL3* — impacted secretion and reabsorption by the nephrocyte. In our colocalization analysis, we identified a single SNP associated with both *MYL3* and *PTH1R* expression and UACR. Interestingly, only *MYL3* knockdown impacted nephrocyte function, providing support for the role of *MYL3* (vs. *PTH1R*) in kidney function. We selected follow-up genes independent of their predicted horizontal pleiotropic effects. Interestingly, we found that all six genes predicted to be pleiotropic did not impact *Drosophila* nephrocyte function (Fig. 6).

The eQTLs augmented by cell-type CREs make the results of downstream analyses (colocalization, PTWAS) interpretable in terms of (1) mechanistic insight into transcriptional regulation and (2) contributing cell-types or *cis-*regulatory elements (Figs. 5, 6). The clinical implication of such interpretable eQTLs can be inspected by the researchers with reduced false-positive hits arising from neutral variants in LD with causal variants. To facilitate secondary analyses for end users, we provide interactive visualizations that include eQTL summary statistics along with cell-type CREs and deltaSVM scores at www.nephqtl2.org (see supplement for tutorial). This portal will be a novel resource to narrow down potential mechanisms and elucidate the regulatory landscape of kidney phenotypes.

The current study is limited in several ways: (1) Although we maximized the discovery of open chromatin peaks of rare cell types, the capability of peak discovery is still limited by the mappability of ATAC-seq reads, which depends on absolute cell counts. By harmonizing this data with data from future assays, we will be able to increase the cell counts and enhance the sensitivity of peak calling. (2) fastENLOC analysis, as well as other colocalization methods, tend to yield few highly confident findings. This is partially because the GWAS and the eQTL data are from different cohorts (the two-sample design), and their LD patterns do not match exactly. In addition, when working with summary statistics, an LD matrix from a third orthogonal population is used, which may not perfectly match the GWAS and eQTL datasets. However, while LD pattern mismatches reduce enrichment estimates and power, false positives are rare^41^. Additionally, the comparison of colocalized loci with previous studies is imperfect due to the use of different methods. (3) Our eQTL dataset was built from a heterogenous nephrotic syndrome cohort from multiple ancestries. While this may allow us to improve fine-mapping^14^ and capture disease-specific eQTLs, the heterogeneity had to be properly controlled. To this end, we adjusted our eQTL analysis with PEER factors, which account for hidden technical and biological structure, and principal components, which account for population stratification. Interpretation of the eQTLs should take these factors into account. Of note, we found strong concordance of our eQTL effect sizes with compartment-matched eQTLs from healthy European samples^23^ (Supplementary Fig. 1).

## Methods

This research was conducted with the informed consent of all study participants and had ethical approval from by the NEPTUNE study and the IRBs at Boston Children’s and Washington University.

For a detailed list of all data and tools used, see Supplementary Data 12.

### Analysis of snATAC/snRNA-seq data

We used our optimized kidney CRE maps generated from a previous study^17^ using publicly available human kidney snATAC-seq data from non-tumor kidney cortex samples from 5 patients undergoing partial or radical nephrectomy (GSE151302)^49^. Briefly, our optimized pipeline for snATAC-seq data processing includes (1) preprocess and quality control of reads with the Cell Ranger ATAC pipeline (v1.1.0) with default options, (2) harmonizing samples with Harmony (v0.1)^50^, (3) cell QC, clustering and type identification with snapATAC (v1; 2019-09-19)^51^, (4) *post hoc* peak calling and optimization in a cell-type resolved manner with MACS2 (v2.2.7.1)^52^ and gkmQC (v1.0)^17^. Specifically, we sorted out cells based on read counts (10^3.5^ ≤ UMI ≤ 10^5^) and the fraction of promoter reads (0.1≤ FRiP ≤ 0.6) with excluding cells in a cluster of potential doublets. A total of 35,286 cells were analyzed to call the peaks. Regarding snRNA-seq, we downloaded count matrices and cell-type labels for the five snRNA-seq samples (GSE151302) to measure gene expression and the cell type identification of snATAC-seq datasets. Specifically, cells were sorted out using Seurat (v3.0.2; 500 <Features <4000, RNA count <16000, %Mitochondrial genes <0.8, %Ribosomal protein large or small subunits <0.4) as shown in Muto et al. Consequently, we profiled open chromatin peaks and gene expression for 16 known kidney cell types. The list of peaks and UMAP plots are available in the original gkmQC paper^17^.

### Analysis of bulk ATAC- and DNase-seq data

For the kidney bulk ATAC-seq data, we used the processed data obtained from Dr. Chakravarti’s laboratory^18^. We obtained representative ENCODE DNase-seq samples of seven bulk tissues (ENCSR543YPH for kidney, ENCSR141VGA for lung, ENCSR148VUP for HMP, ENCSR272RQX for muscle, ENCSR649KBB for brain, ENCFF354YDR for CMP, ENCSR911LTI for heart^24^) with the best quality chosen by gkmQC^17^. To process bulk DNase-seq and ATAC-seq, we adapted the previously established framework for DNase-seq and bulk ATAC-seq analyses as used in Lee et al.^53^ and Nandakumar et al.^54^. The pipeline includes cutadapt v4.1 for preprocessing reads, Picard 2.26.10 for duplicates removal, bowtie2 v2.4.4 for the alignment with GRCh38, and MACS2 v2.2.4 for calling peaks. We used the same *post hoc* peak calling and optimization as used in snATAC-seq analysis gkmQC^17^.

### Heritability enrichment analysis

We used stratified LD-score regression (S-LDSC; v1.0.1)^19^ to estimate the proportion and enrichment of heritability from GWAS summary statistics. The proportion of the heritability contributed by a SNP set (*C*) in open chromatin peaks from a sample is calculated as follows:

1 $${\Pr }_{{{{{{\rm{C}}}}}}}({h}_{{{{{{\rm{SNP}}}}}}}^{2})={h}_{{{{{{\rm{SNP}}}}}}}^{2}(C)/{h}_{{{{{{\rm{SNP}}}}}}}^{2}$$

The enrichment of proportional heritability then is calculated by Pr_*C*_(*h*^2^)/Pr_*C*_(*M*), where Pr_*C*_(*M*) is the proportion of SNPs in *C* among the total SNP set (M). The standard error and statistical significance of the enrichment is estimated using block jackknife method implemented in the LDSC software^19^. For reference LD scores, European ancestry population and the corresponding allele frequencies in 1000 Genomes Phase 3 data were used (v2.2; https://data.broadinstitute.org/alkesgroup/LDSCORE/). We considered open chromatin regions to be regions that extend ±1 kb from the peak summits^17^, as this includes the set of potentially associated regulatory variants. When comparing multiple functional annotations (e.g., multiple groups of CREs specific for different cell types), we conducted S-LDSC regression jointly with the annotations, along with the full set of the baseline annotations.

### Positive and negative-control phenotypes

To validate the cell-type convergence of heritability/colocalization with kidney GWASs, we repeated the analyses for 7 additional kidney phenotypes^7,29,30^ and GWAS of 19 diseases and traits from the UK Biobank (negative controls)^31^. For negative controls, we selected UKBB phenotypes with insignificant genetic correlation (|*r*_g_| < 0.1, *p* > 0.1; LDSC) and with at least as many significant LD blocks as the lower powered GWAS of UACR (a proxy for power based on EUR LD blocks with at least one SNP having posterior probability > 0.2).

### Kidney RNA-seq

Total RNA from microdissected biopsies (240 GLOM, 311 TUBE) from the NEPTUNE study^16^ were prepared using the SMART-Seq v4 RNA kit (Takara Bio USA, Mountain View, CA, USA). Samples underwent sequencing using Illumina HiSeq 2500, resulting in 150 bp unstranded, paired-end reads. Fastq files underwent quality control filtering and trimming using fastQC (v 0.11.5), fastQScreen (v0.11.4)^55^, and Picard Tools (http://broadinstitute.github.io/picard; v2.4.1). Trimmed reads were aligned to the human genome (GRCh37) with STAR 2.6.0a^56^. Gene expression counts were quantified using StringTie (v2.1.4)^57^. Gene expression was normalized across samples using TMM normalization with edgeR (v3)^58^, only keeping genes with greater than 0.1 cpm (counts per million; ~6 counts) in 20% of the samples. Transformed expression values were then rank-based inverse normalized.

### WGS

Whole genome sequencing (30x) was done using the Illumina HiSeq system. Alignment and variant calling were performed using default settings of GotCloud (v1.12.3) with the GrCh37 reference of the human genome^59^. Variants underwent the following quality control filters using VCFtools (v0.1)^60^, PLINK (v1.9)^61^ and the HardyWeinberg R(v3.5.1) package^62^: multi-allelic variants were converted to bi-allelic, variants with GQ < 20 and AB < 0.2 or > 0.8 were set to missing, variants with genotyping rate <0.85, MAF < 0.01 and inbreeding coefficient < −0.3 were removed, and variants failing HWE, *p* < 10^−6^, in either European or African subsamples were removed. As a proxy for population stratification, we calculated principal components in PLINK using LD-pruned WGS.

### Single-SNP eQTL analysis

Single-SNP*cis*-eQTL ( ± 1 Mb) analysis was performed with MatrixEQTL (v2.3)^20^ adjusting for age, sex, batch, 4 genotype PCs, and PEER factors (v1.3)^63,64^. The optimal number of PEER factors was selected based on the maximum number of eGenes, as determined by TORUS^26^, resulting in a variable number of PEER factors. Of note, the single-SNP eQTL results were used to estimate enrichment of the CRE annotations in TORUS and were not used for eGene calling. We validated our eQTLs by comparing the effect size and direction of associations and not the overlap of eGenes, since calling of eGenes can be influenced by sample size and methods. Pearson correlation of SNP effect sizes for the top-ranked 5,000 genes to other eQTL analyses including GLOM and TUBE from Gillies et al.^21^ and Qiu et al.^23^ and GTEx kidney cortex^46^. To globally compare our GLOM and TUBE analyses to all GTEx tissues, we calculated principal components of *z*-scores from GLOM, TUBE and GTEx V8 eQTL analyses. For each gene, the SNP with the largest *z*-score across all studies was selected, resulting in one strong SNP association per gene.

### Enrichment of eSNPs in CREs of relevant kidney cell and tissue types

Enrichment of epigenetic annotations was estimated using TORUS^26^ excluding the distance to TSS annotation. For this analysis, the SNP annotations were generated for each cell type. To compare the enrichment of eSNPs in CREs across different cell types, we controlled several potential confounding factors. First, we used peaks called from the same number of subsampled cells (*N* = 300). Second, we used the results of MatrixEQTL from the matched samples (*N* = 219) between GLOM and TUBE. To analyze the baseline level of enrichment scores, we constructed two different matched control sets for the cell-type peaks: (1) peaks with the same number of target cell-type peaks randomly selected from the union of peaks covered in our kidney CRE map, and (2) randomly chosen genomic regions that have similar GC-contents and repeat fractions with the target cell-type peaks. To compare with different tissues, we applied our (optimized) pipeline to call the peaks from ENCODE DNase-seq datasets.

### Enrichment of eGene expression in relevant kidney cell and tissue types

We binarized snRNA-seq gene expression (GSE151302), where genes with non-zero expression in at least 2% of the cells were considered expressed for a given cell type. We performed a permutation analysis to compute cell-type specific gene enrichment of the top 5000 MatrixEQTL eGenes. To generate the null distribution, we randomized gene expression for each cell type, assuming a uniform distribution, and calculated the number of MatrixEQTL eGenes expressed for each cell type, repeating 1000 times. We calculated a *z*-score to quantify cell-type-specific gene enrichment in the snRNA-seq data set.

We conducted a similar permutation analysis using GTEx bulk RNA-seq data V8 obtained from the GTEx portal. To binarize gene expression, we ranked genes by median gene-level TPM for each tissue and classified genes ranked within the top 5000 as being expressed.

### SNP prior generation

Cell-type enrichment (“Methods”: Enrichment of eSNPs in CREs of relevant kidney cell and tissue types and Enrichment of eGene expression in relevant kidney cell and tissue types) and biological relevance were considered when selecting cell-types to use for each tissue prior.

Integrative prior: We use two SNP annotations to generate the integrative prior: (1) distance of the SNP to the given genes TSS (which gets binned in TORUS), and (2) binary indicator for the SNP being in open chromatin (from the union of tissue-relevant cell types). For GLOM and TUBE separately, we generated genome-wide base-pair resolution annotation files, where SNPs within ±300 bp from a summit of ATAC-seq peaks from the union of selected cell-types (pseudo-bulk) were coded with a binary indicator (within peaks=1, outside peaks=0). Using TORUS and the single-SNP results from MatrixEQTL, we calculated enrichment of the CRE annotation jointly with the enrichment of the distance to the given gene’s TSS using default setting. The enrichment estimates are then used to weight SNPs appropriately and generate SNP priors for each gene. Given the positive enrichment of our annotations, SNPs closer the TSS and SNPs in open chromatin have higher prior probabilities than other SNPs.

The following priors were generated for comparative purposes only:

TSS prior: includes only the distance to the TSS, not the CRE annotations.

Uniform prior: All SNPs are equally probable and have identical weights.

### Multi-SNP eQTL analysis

We performed our integrative eQTL analyses with Deterministic Approximation of Posteriors (DAP-G)^27,28^ using genotype and expression data from NEPTUNE and each of the priors (uniform, TSS, and integrative) generated by TORUS (Methods: SNP Prior generation). We adjusted for PEER factors (40 in GLOM, 50 in TUBE), age, sex, 4 genotype PCs, and RNA-seq batch. Using the genotype data to calculate LD, eQTL clusters were formed with SNPs in a cluster having an R^2^ > 0.25. Gene-level Bayesian FDR methods were used to identify eGenes in each tissue and 95% credible sets were formed by summing ranked SNPs for each gene cluster. The uniform and TSS priors are only for comparative purposes; results on nephqtl2.org are from the integrative prior.

### eQTL replication analyses

To evaluate the increase in eGene discovery (independent of increased sample size and different samples) and assess fine-mapping replication, we conducted multiple eQTL analyses by splitting GLOM and TUBE samples into two independent subsets each. Our discovery samples consisted of samples used for NephQTL (Gillies et al.), and our replication analysis included all other samples (N_GLOM_discovery_ = 96, N_GLOM_replication_ = 144, N_TUBE_discovery_ = 122, N_TUBE_replication_ = 189). For each group, we conducted eQTL analysis as described above. Overlap of eGenes was assessed with upset plots. To compare prioritization of fine-mapped SNPs, we first identified all significant clusters with eGene FDR < 0.05 and cluster posterior probabilities > 0.2 in the discovery analyses. SNPs from each cluster were then matched with the replication analysis. Spearman rank correlation was used to compare SNP rankings between analyses. To show the increase in SNP prioritization with our integrative prior, we compared SNP rank correlations from fine-mapping with DAP to Spearman rank correlations of SNPs from single-SNP eQTL analysis with MatrixEQTL. The distribution of rank correlations was compared with a Wilcoxon rank sum test.

### Comparison of fine-mapping results from different priors

We compared properties of the 95% credible sets to quantify fine-mapping resolution. For each credible set generated by each prior, we identified (1) the maximum snpPIP and (2) the number of SNPs in the credible set. Distributions from the uniform and integrative prior were compared with Wilcoxon rank sum tests.

### Computational prediction of regulatory effects

We generated deltaSVM scores to computationally predict the functional impact of SNPs^65^. Open chromatin peaks of each cell type were used as a positive training set to build gkm-SVM models^66^ as previously described, with some modifications. The LS-GKM (v0.1.1)^67^ software with default parameter settings was used for training. To calculate comparable scores across cell-type models, (1) the top 100,000 peaks were used to train each model, and (2) deltaSVM scores were normalized per cell type using *z*-score based normalization of the distribution created by common SNPs with MAF > 1% in European ancestry from the 1000 Genemies project^68^. *Z*-scores were transformed to probability scores for being functional variants using a logistic model trained by dsQTLs of lymphoblastoid cell line (LCL)^69^ as a positive set and random SNPs with the control of GC contents and repeat fractions as a negative set. To aggregate the deltaSVM scores for GLOM and TUBE, we used the transformed scores of SNPs in peaks of the corresponding cell type and chose the best score per SNP among cell types whose CREs were used as the prior of corresponding eQTLs. We regarded the SNPs with aggregated score >0.5 as deltaSVM-positive in GLOM or TUBE compartments.

To test the significance of deltaSVM scores of the lead eSNPs, we identified random SNPs controlling for allelic frequency, distance from TSS, and the signal strength of open chromatin peaks (i.e., signal value of peak calling derived from MACS2). For per-SNP random control, we allowed 1%, 1000 bp, and 1.0 as the residual error of the corresponding controlling variables, respectively.

### Colocalization

To test for colocalization of phenotype-associated SNPs and eSNPs from our tissue-specific eQTL analysis, we used fast enrichment estimation aided colocalization analysis, fastENLOC (v1.0), with default settings. The fine-mapped DAP results were converted to vcf format using the provided script summarize_dap2enloc.pl. *Z*-scores were extracted from trans-ethnic GWAS summary statistics (eGFR/UACR)^6,7^ and European 1000 Genomes project phase 3 version 5 samples^68^ were used for the LD reference panel in all analyses. Of note, colocalization analysis with the European-only GWAS summary statistics yielded similar results.

To assess enrichment of colocalized SNPs in our CRE annotations used in the integrative priors, we expanded our colocalization analyses to multiple GWAS of primary kidney and UK Biobank phenotypes (see “Positive and negative-control phenotypes” above for phenotype selection). Colocalization analysis was conducted as described above for each GWAS with both GLOM and TUBE eQTLs and the uniform and integrative prior. For each GWAS-eQTL-prior pairing, we identified colocalized loci with RCP ≥ 0.2. For each cluster, we selected a single SNP with the highest colocalization probability. When there were multiple top SNPs, we prioritized SNPs that were in both the uniform and integrative prior and SNPs within CRE annotations. To focus on the effect of each prior on colocalization, we removed loci that prioritized the same top SNP independent of prior selection. To generate the expected number of SNPs within CRE annotations for each analysis, we randomly selected SNPs (100 × number of colocalized SNPs) controlling for the rank of the colocalized SNPs in the eQTL model. We calculated the mean overlap from 10 simulations for each GWAS-eQTL-prior pairing. The enrichment significance was tested with a one-sided Binomial test of the observed overlap using the estimated expectations.

### Transcriptome-wide association analysis

Probabilistic transcriptome-wide association analysis (PTWAS; v1.0^36^) was used to test for causal relationships between GLOM and TUBE gene expression and complex kidney phenotypes—trans-ethnic meta-analyses of UACR^6^ and eGFR^7^. Using the fine-mapped DAP results, glomerular and tubulointerstitial eQTL gene-SNP weights were calculated and formatted for GAMBIT gene-based testing using PTWAS helper scripts provided by the program authors (ptwas_builder, make_GAMBIT_DB.R). 1000 Genomes project phase 3 version 5 samples were used for the LD reference panel in all analyses. PTWAS_scan was run using default settings. Gene-level significance was adjusted to account for the multiple testing burden using two methods; *q*-value^70^ and a more conservative Bonferroni threshold. Genes with predicted pleiotropic effects or no strong instruments were excluded from our count of significant loci. We tested for pleiotropic effects and estimated effect sizes using the ptwas_est function.

### Gene validation in *Drosophila* nephrocytes

Three young female *Drosophila melanogaster* (common fruit flies, hatched within three days) were used in this experiment. Ethical approval was not needed given no vertebrate animals were used. Genes for functional validation were selected based on the following criteria (1) causal association between gene and kidney phenotype in PTWAS analysis (FDR ≤ 0.05); (2) colocalization of eSNP associated with eGene and GWAS variants (RCP ≥ 0.5); (3) colocalization of eSNPs and relevant cell-type open chromatin; and (4) relative high expression levels of *Drosophila* homologs in the nephrocyte. A random subset of the qualifying genes was selected for functional follow up.

#### ANF‑RFP uptake assay

Briefly, 10 virgin female flies from the MHC-ANF-RFP, Hand-GFP and Klf15-Gal4 transgenic lines were crossed to 5 male flies from UAS-RNAi transgenic lines of the targeted genes at 25 °C. Pericardial nephrocytes of newly emerged adult flies (within 24 h of eclosion) were dissected and kept in artificial *Drosophila* hemolymph to assay RFP accumulation detected by fluorescence microscopy. For quantification of relative ANF-RFP fluorescence, 20 nephrocytes were analyzed from each of 3 flies per indicated genotype. T-tests were used to indicate significance differences from the control.

#### Dextran uptake assay

Flies carrying Hand-GFP and Klf15-Gal4 transgenes were crossed with flies carrying the UAS-RNAi transgenes at 25 °C. Dextran uptake was assessed in adult flies one-day post-emergence by dissection of pericardial nephrocyte in artificial *Drosophila* hemolymph and examination of the cells by fluorescence microscopy after a 20 min incubation with Texas Red labeled Dextran (Thermo Fisher, cat# D1828; 10 kD, 0.02 mg/ml). For quantification of relative Dextran dye fluorescence, 20 nephrocytes were analyzed from each of 3 flies per indicated genotype. T-tests were used to indicate significance differences from the control.

### Luciferase reporter assay for allele-specific enhancer activity of rs80282103

Approximately 500-bp regions of DNA containing rs80282103 and rs11154336 were amplified from purified human genomic DNA (Promega, #G1521) by PCR using engineered restriction sites to allow directional cloning into the multiple cloning region of the pGL4.23[luc2/minP] luciferase reporter vector (Promega, #E841A). The resulting plasmids containing the insert in either forward or reverse orientation were confirmed by Sanger sequencing. Constructs containing the alternate alleles were obtained by performing Q5 site-directed mutagenesis (NEB, #E0554S). Primers used to amplify targets and perform site-directed mutagenesis are listed in Supplementary Data 11. Each luciferase construct was co-transfected with pGL4.74[hRluc/TK] vector (Promega, #E692A), a *Renilla* luciferase control reporter, in HK-2 human proximal tubule cells (American Type Culture Collection [ATCC], #CRL-2190) cultured in DMEM/F-12 (Gibco, #11320033) supplemented with 10% FBS (Gibco, #10437028) at approximately 70% confluency in 96-well plates by using TransIT-2020 Reagent (Mirus, #5404), following the manufacturer’s protocol. Three separate transfections were performed with four technical replicates in each plate. Empty luciferase vector, pGL4.23[luc2/minP], was also transfected in quadruplicate as a control. Luciferase activity was quantified 48 hours after transfection using the Dual-Glo Reporter Assay System (Promega, #E2920) according to the manufacturer’s protocol. Luminescence signals were captured using a GloMax®-Multi+ Detection System (Promega) and normalized to *Renilla* luciferase readings for each well. We used linear regression with log-transformed normalized luminescence adjusting for batch and orientation to test the allele effect on enhancer activity.

### Statistical analyses and visualization

R 3.5 and Python 3.7 with 3rd-party package (scipy) were used to perform statistical analysis. IGV (2.12.3), LocusZoom, ggplot2 were used for visualizing open chromatin, GWAS, eQTL datasets.

### Reporting summary

Further information on research design is available in the Nature Portfolio Reporting Summary linked to this article.

## Supplementary information

Supplementary Information

Description of Additional Supplementary Files

Supplementary Data 1-12

Reporting Summary

## Data Availability

Raw data used to generate results are available through NEPTUNE (https://www.neptune-study.org/ancillary-studies). The processed data of kidney fine-mapped eQTL and chromatin accessibility analysis are publicly available online at the NephQTL2 (https://www.nephqtl2.org). We utilized GotCloud with the hg19 resource files available at https://genome.sph.umich.edu/wiki/GotCloud:_Genetic_Reference_and_Resource_Files. Single-cell ATAC-and RNA-seq datasets were downloaded from GEO website (GSE151302). Bulk ATAC-seq data (human kidney samples) is from Dr. Chakravarti’s laboratory & Lee et al., 2022 (https://www.biorxiv.org/content/10.1101/2022.04.19.488795v1.abstract). Bulk DNase-seq data from ENCODE (ENCSR543YPH) for kidney, ENCSR141VGA for lung, ENCSR148VUP for HMP, ENCSR272RQX for muscle, ENCSR649KBB for brain, ENCFF354YDR for CMP, ENCSR911LTI for heart. Bulk RNAseq and eQTL tissue-specific all SNP gene associations were downloaded from the GTEx consortium (https://storage.googleapis.com/gtex_analysis_v8/rna_seq_data/GTEx_Analysis_2017-06-05_v8_RNASeQCv1.1.9_gene_reads.gct.gz & https://console.cloud.google.com/storage/browser/gtex-resources). Datasets used to compare eQTL effect sizes were downloaded from https://nephqtl.org/, The Susztak Lab (https://susztaklab.com/Kidney_eQTL/download.php), and GTEx (https://console.cloud.google.com/storage/browser/gtex-resources). Summary statistics of eGFR/UACR GWAS were downloaded from CKDGen Consortium (https://ckdgen.imbi.uni-freiburg.de), GWAS catalog (https://www.ebi.ac.uk/gwas/studies/GCST90100220), and UK Biobank (https://pan.ukbb.broadinstitute.org/downloads/index.html & https://docs.google.com/spreadsheets/d/1AeeADtT0U1AukliiNyiVzVRdLYPkTbruQSk38DeutU8/edit#gid=268241601). Datasets used in the analysis are also outlined in Supplementary Data 12.

## Code availability

All analyses were conducted with publicly available software. A detailed list of tools can be found in Supplementary Data 12.
