# Supplementary material for: Mapping genomic regulation of kidney disease and traits through high-resolution and interpretable eQTLs
Source: Nat Commun. 2023 Apr 19;14:2229. doi: 10.1038/s41467-023-37691-7 (PMC10115815; doi:10.1038/s41467-023-37691-7)
Supplement: Supplementary file 1 — Supplementary Information [file 41467_2023_37691_MOESM1_ESM.pdf]

This PDF includes:

Supplementary Figs. 1-12 (Pages 2-18)

NephQTL2.org Tutorial (Pages 19-25)

NEPTUNE Consortium Authors (Page 26)

Supplementary Data 1-12 are available in a separate Excel file.

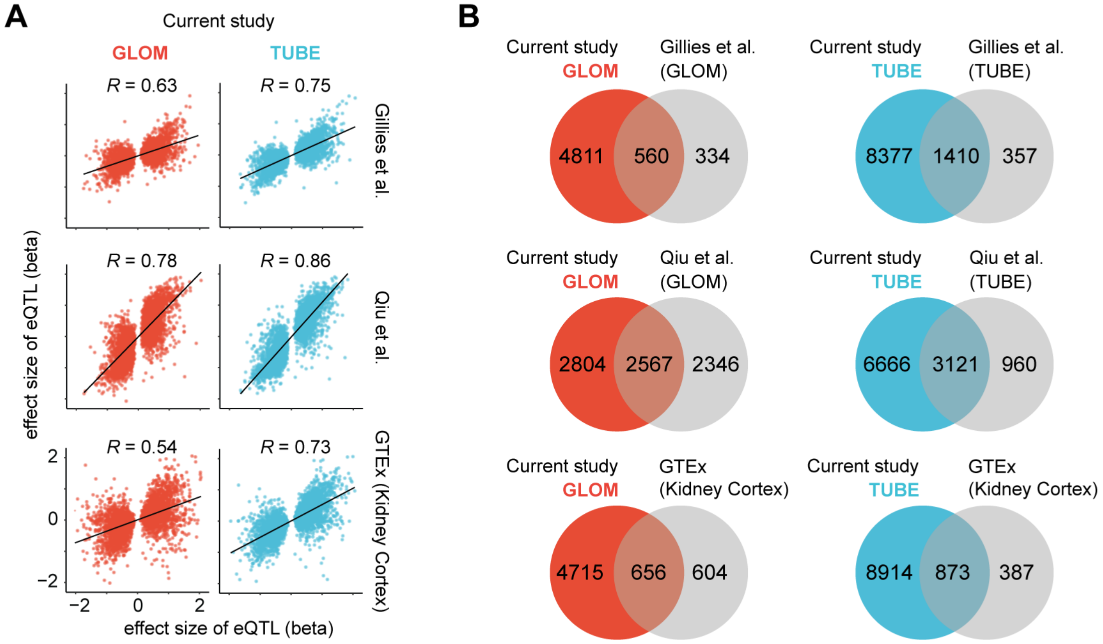

Supplementary Figure 1. **Concordance of eQTLs across publicly available eQTL datasets**. **A** Scatter plot and correlation of the effect sizes of GLOM and TUBE eQTLs paired with each kidney eQTL dataset. **B** Venn diagrams showing overlap of eGenes between current and other studies.

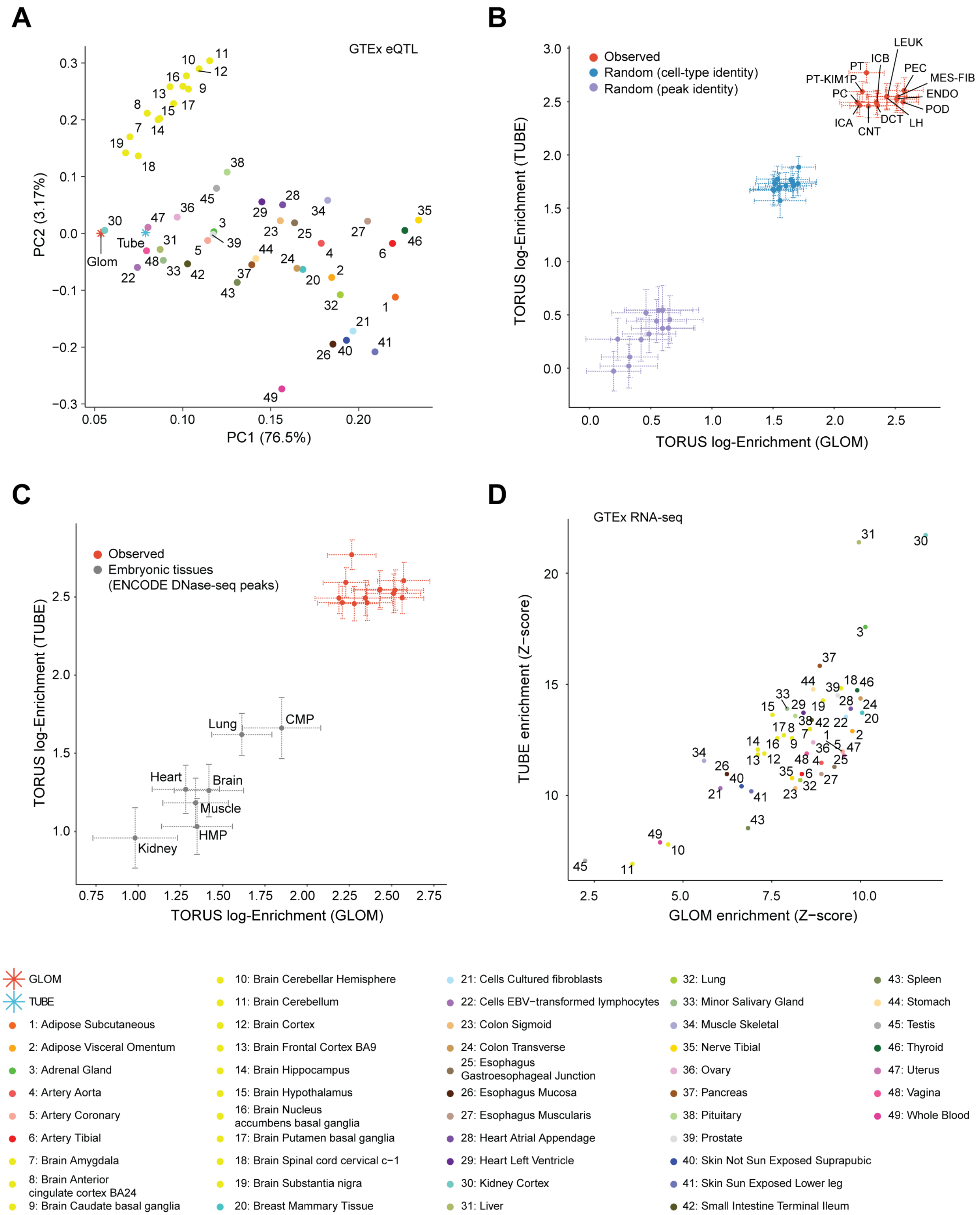
A
GTEx eQTL
PC2 (3.17%)
PC1 (76.5%)
Glom
Tube
B
Observed
Random (cell-type identity)
Random (peak identity)
LEUK
ICB
PT
PEC
PT-KIM1P
MES-FIB
PC
ENDO
POD
DCT
LH
ICA
CNT
TORUS log-Enrichment (TUBE)
TORUS log-Enrichment (GLOM)
C
Observed
Embryonic tissues
(ENCODE DNase-seq peaks)
Lung
CMP
Heart
Brain
Muscle
HMP
Kidney
TORUS log-Enrichment (TUBE)
TORUS log-Enrichment (GLOM)
D
GTEx RNA-seq
TUBE enrichment (Z−score)
GLOM enrichment (Z−score)
GLOM
TUBE
1: Adipose Subcutaneous
2: Adipose Visceral Omentum
3: Adrenal Gland
4: Artery Aorta
5: Artery Coronary
6: Artery Tibial
7: Brain Amygdala
8: Brain Anterior cingulate cortex BA24
9: Brain Caudate basal ganglia
10: Brain Cerebellar Hemisphere
11: Brain Cerebellum
12: Brain Cortex
13: Brain Frontal Cortex BA9
14: Brain Hippocampus
15: Brain Hypothalamus
16: Brain Nucleus accumbens basal ganglia
17: Brain Putamen basal ganglia
18: Brain Spinal cord cervical c−1
19: Brain Substantia nigra
20: Breast Mammary Tissue
21: Cells Cultured fibroblasts
22: Cells EBV−transformed lymphocytes
23: Colon Sigmoid
24: Colon Transverse
25: Esophagus Gastroesophageal Junction
26: Esophagus Mucosa
27: Esophagus Muscularis
28: Heart Atrial Appendage
29: Heart Left Ventricle
30: Kidney Cortex
31: Liver
32: Lung
33: Minor Salivary Gland
34: Muscle Skeletal
35: Nerve Tibial
36: Ovary
37: Pancreas
38: Pituitary
39: Prostate
40: Skin Not Sun Exposed Suprapubic
41: Skin Sun Exposed Lower leg
42: Small Intestine Terminal Ileum
43: Spleen
44: Stomach
45: Testis
46: Thyroid
47: Uterus
48: Vagina
49: Whole Blood

Supplementary Figure 2. The comparison of GLOM/TUBE eQTLs with eSNP/Genes and eQTL-association from GTEx/ENCODE samples. A Principal component analysis of *z*-scores from independent eQTLs in GLOM, TUBE and GTEx tissues **(Methods). B** Red dots depict observed enrichment of our snATAC-seq peaks. Blue dots depict the enrichment of eSNPs in randomly sampled peaks from the union set of peaks across kidney cell types. Purple dots depict the enrichment of eSNPs in random regions of human genome (off peaks) that have the similar GC-contents and repeat fractions with the peaks of kidney cell types. Error bar depicts 95% confidence intervals of the enrichment estimate calculated by TORUS (**Methods**). **C** Enrichment analyses of eSNPs in open chromatin peaks of embryonic ENCODE tissues. **D** Enrichment analysis of eGenes in genes expressed in GTEx tissues. POD podocyte, PEC parietal epithelial, MES-FIB mesangial+fibroblast, ENDO endothelial, LEUK leukocytes, PT proximal tubule, LH loop of Henle, DCT/CNT distal convoluted / connecting tubule, PC principal cells, ICA/ICB type A/B intercalated cells, PT-KIM1+ KIM1 positive proximal tubule.

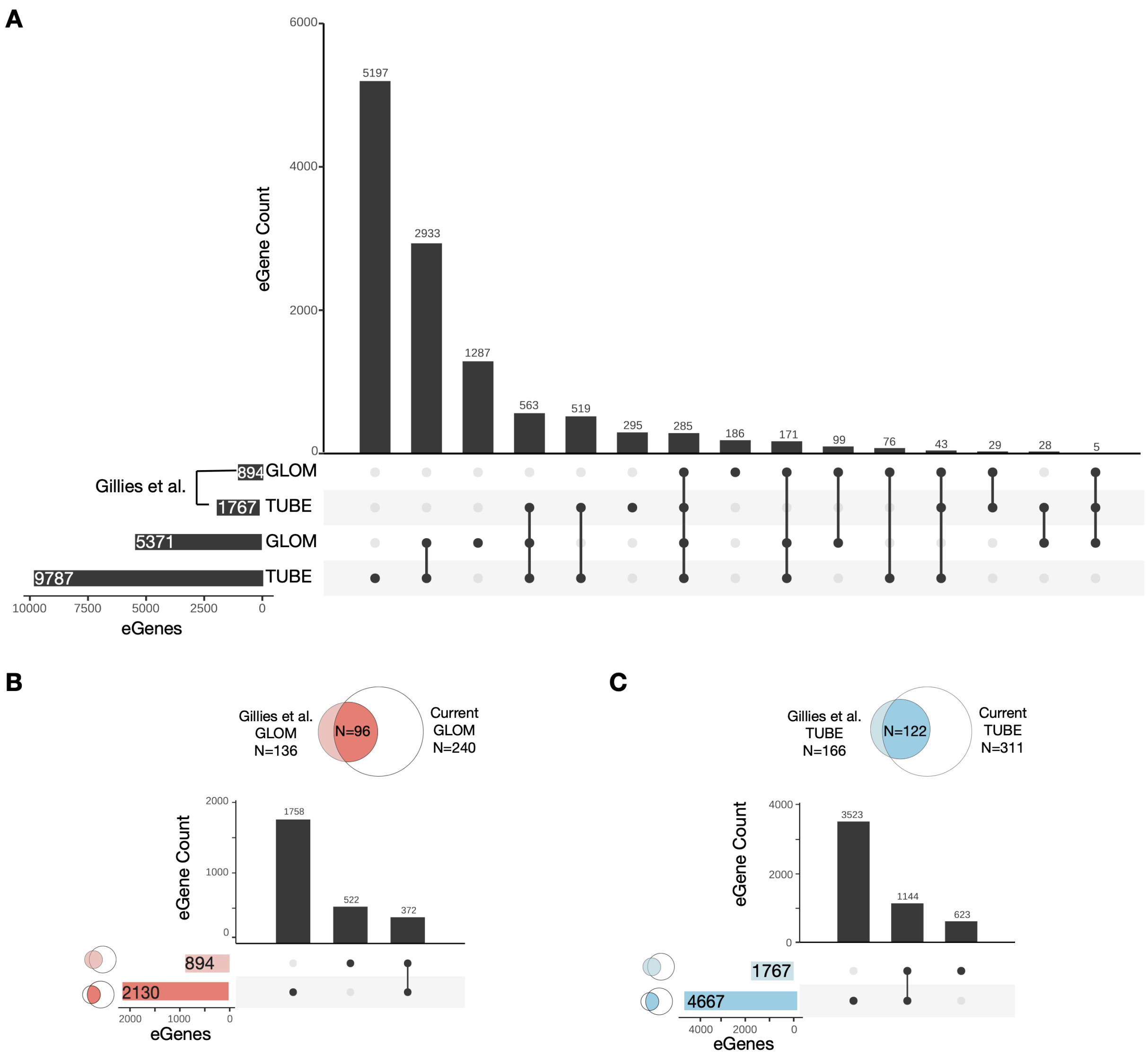

Supplementary Figure 3. UpSet plots comparing overlap of eGenes from the current study to array-based eQTL analyses. A Total significant eGenes from the high-resolution CRE-informed GLOM and TUBE eQTL analyses compared to the total significant eGenes from the previous, array-based eQTL study (Gillies et al., 2018). On the left, the total set sizes for GLOM and TUBE in each study are represented by the horizontal bar graphs. Each vertical bar graph corresponds to the number of eGenes within each possible intersection of the four datasets. For both **B** GLOM and **C** TUBE, UpSet plots comparing overlap of significant eGenes when only the subset of matched samples present in the current study and Gillies et al. study (n=96 in GLOM, n=122 in TUBE) were used to compute high-resolution eSNPs and eGenes. Significant eGenes are defined using gene-level Bayesian FDR cut-off of 0.05.

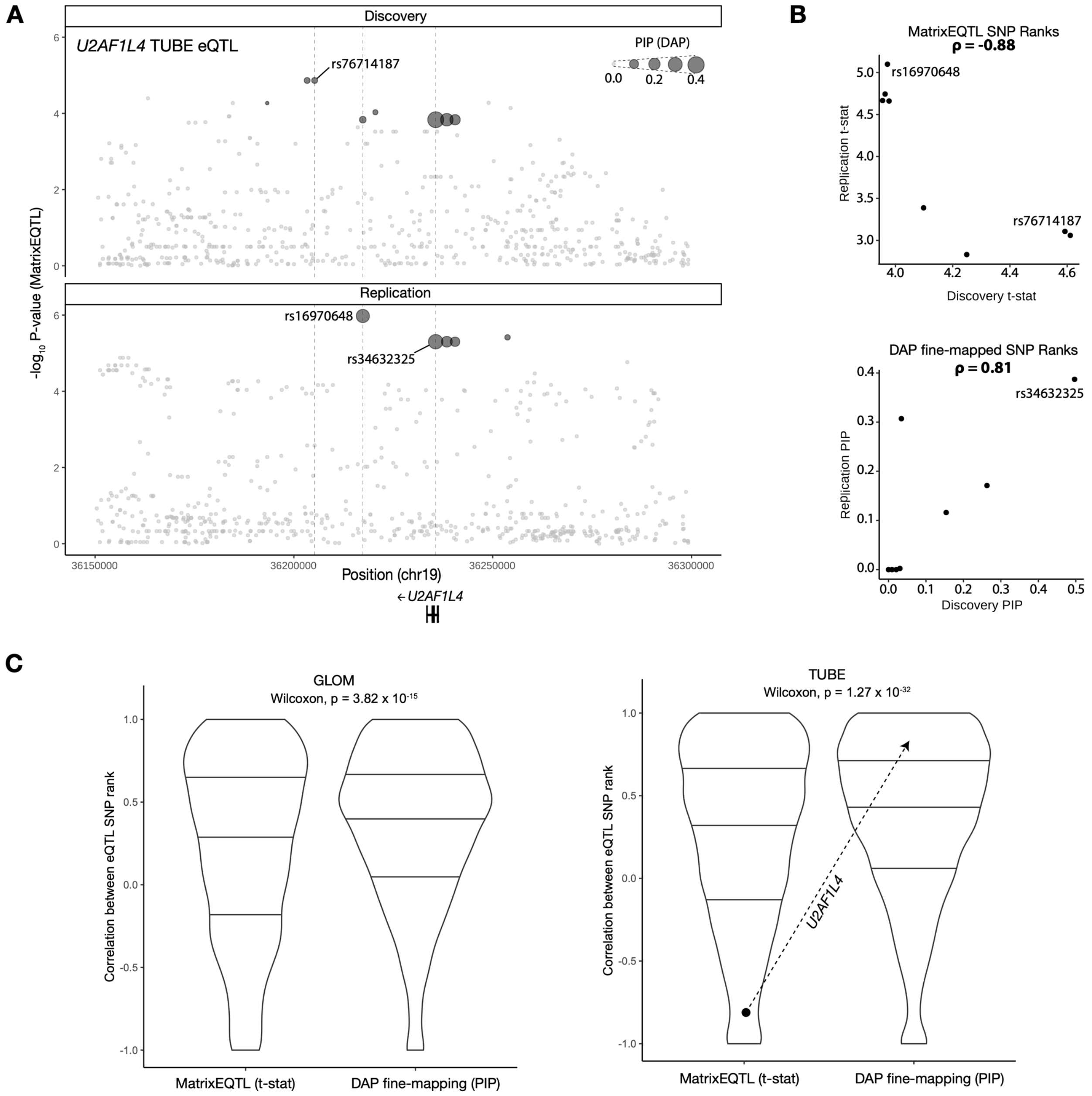

Supplementary Figure 4. Correlation of fine-mapped SNP ranks. A TUBE eQTL Manhattan plot for *U2AF1L4* in the discovery (N=122) and replication (N=189) sample subsets. This gene was selected for illustrative purposes. *P*-value is from single-SNP analysis with MatrixEQTL, the point size is posterior inclusion probability (PIP) from DAP, and dark gray SNPs are included in the eQTL cluster (R^2 > 0.25). Three top SNPs are labeled with rsID and vertical dashed lines. **B** The Spearman rank correlation of two-

sided t-statistics from MatrixEQTL (top) and PIPs from fine-mapping with DAP (bottom). All SNPs clustered in the discovery analysis were included. Top SNPs in each panel are labeled. **C** Comparison of SNP rank correlations across all eGene clusters (cluster PIP > 0.25) for GLOM (left) and TUBE (right). Unadjusted, two-sided Wilcoxon rank sum tests were used to test the difference between correlation distributions. The change in SNP rank correlation from the *U2AF1L4* example from **A** and **B** is highlighted.

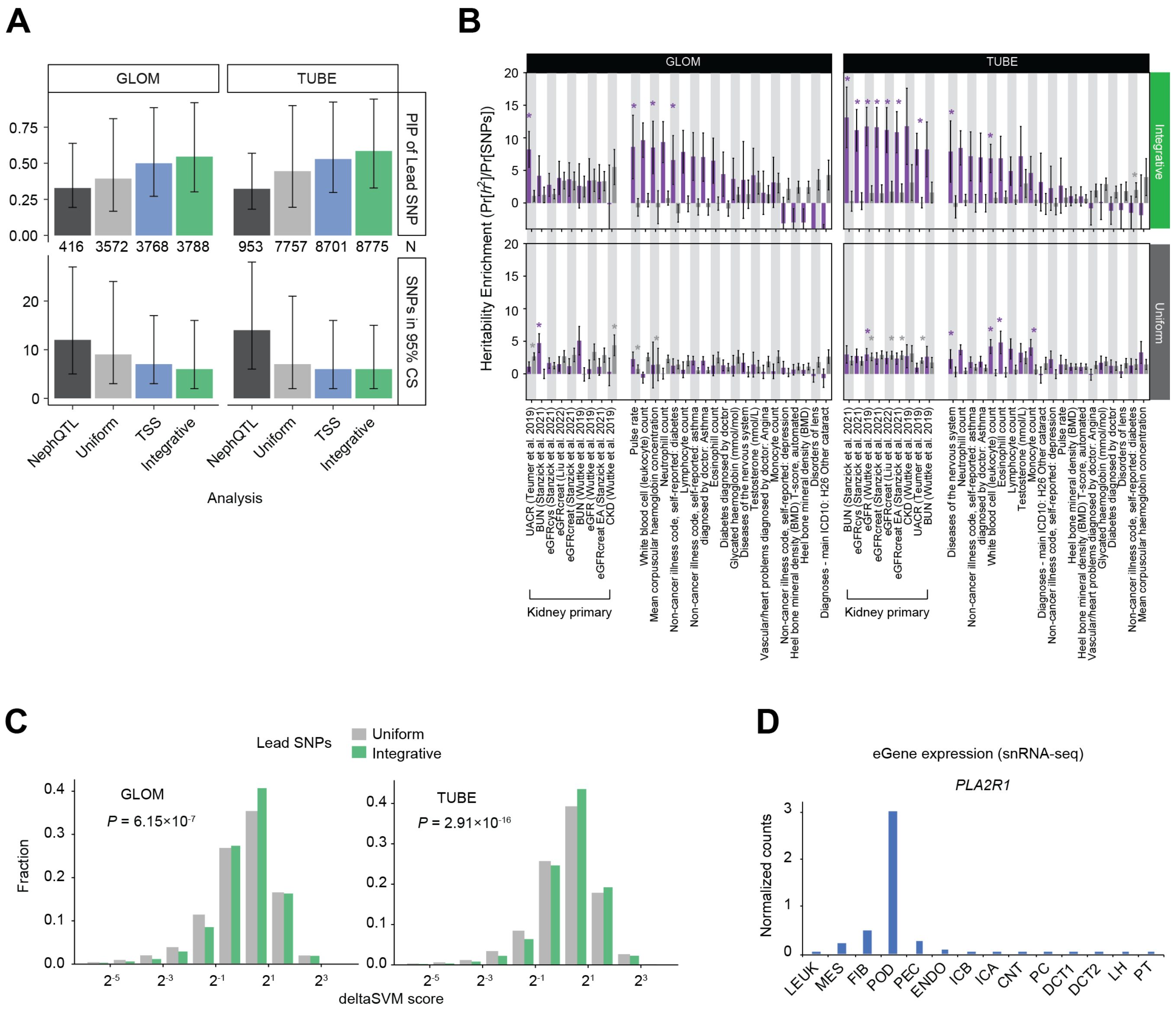

Supplementary Figure 5. Comprehensive analyses of fine-mapping quality across different priors. **A** The distribution of the top SNPs' snpPIPs and number of SNPs forming the 95% credible set. Boxes indicate median and bars are interquartile range (IQR). Compared to **Figure 4A**, NephQTL (Gillies et al., 2018; TSS-only prior) and current eQTLs with TSS-only informed prior are added. N = the number of credible sets included in each analysis. **B** S-LDSC enrichment analysis using the GWAS of kidney primary phenotypes and UK-biobank phenotypes not significantly genetically correlated with eGFR/UACR. For each disease/trait, heritability enrichment was measured using the uniform prior (bottom panels) and the integrative prior (upper panels). Error bar and asterisk depict standard error and the significance of the enrichment estimated using block jackknife method. *Stratified LD-score regression

(S-LDSC) P ≤ 0.05. Exact P-values are in Supplementary Data 6. **C** Comparisons of deltaSVM scores between lead SNPs from uniform and integrative priors as shown in **Figure 4C**. *P*-value is calculated from the two-sided Wilcoxon rank-sum test. **D** Gene expression of *PLA2R1* in snRNA-seq data normalized by genes and cell counts.

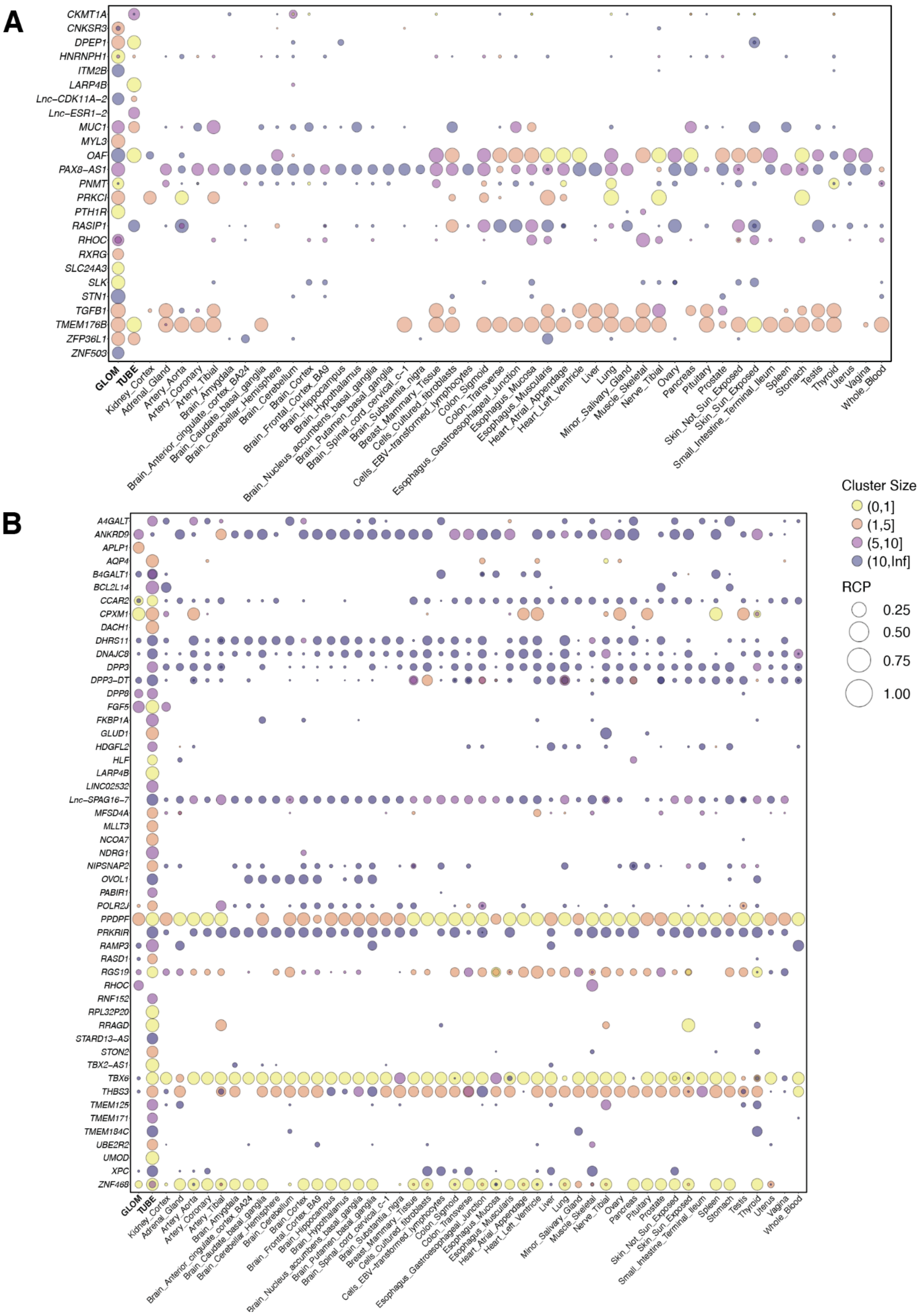
A
B
Cluster Size
(0,1]
(1,5]
(5,10]
(10,Inf]
RCP
0.25
0.50
0.75
1.00
GLOM
TUBE

Supplementary Figure 6. Colocalized genes using integrative prior. Each bubble plot shows GLOM and/or TUBE eGenes colocalized with **A** UACR and **B** eGFR GWAS loci (≥1 cluster with Regional colocalization probability (RCP) ≥ 0.5) along with GTEx colocalization results. Each circle represents an eQTL cluster, and its diameter and color depict the RCP scores and the cluster size (number of eSNPs), respectively.

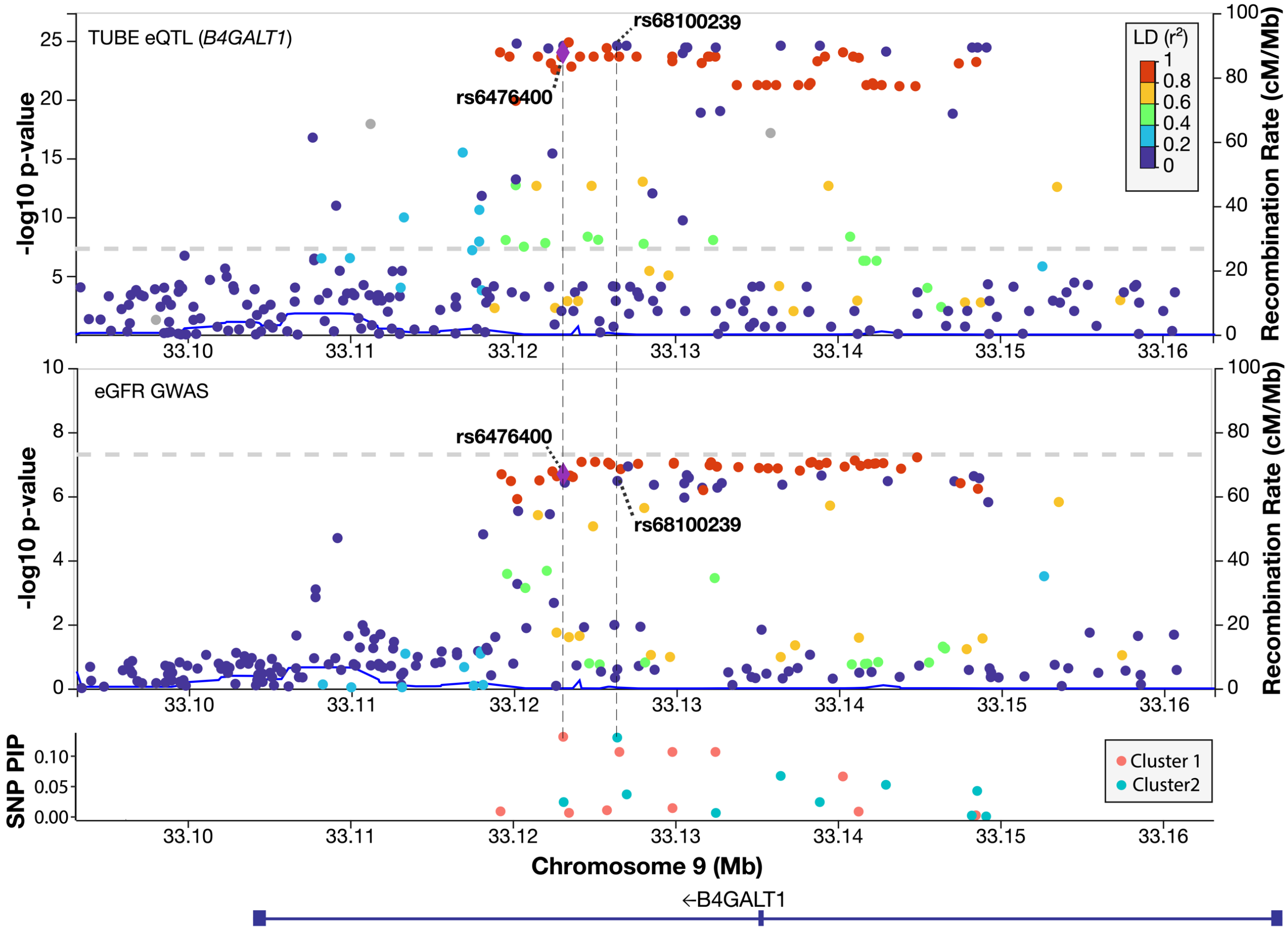

Supplementary Figure 7. eGFR GWAS and TUBE eQTL colocalization at *B4GALT1*. Manhattan plots highlighting two independent colocalized signals within the *B4GALT1* locus: eQTL (top), eGFR GWAS (middle), and colocalization posterior inclusion probability (PIP; bottom). Each labeled SNP is the top SNP for its cluster. LD reference includes all 1000 Genomes populations, with R^2 = 0.06 between rs6476400 and rs68100239. Each SNP is tagging an independent colocalized eGFR and TUBE eQTL association as annotated in the bottom panel.

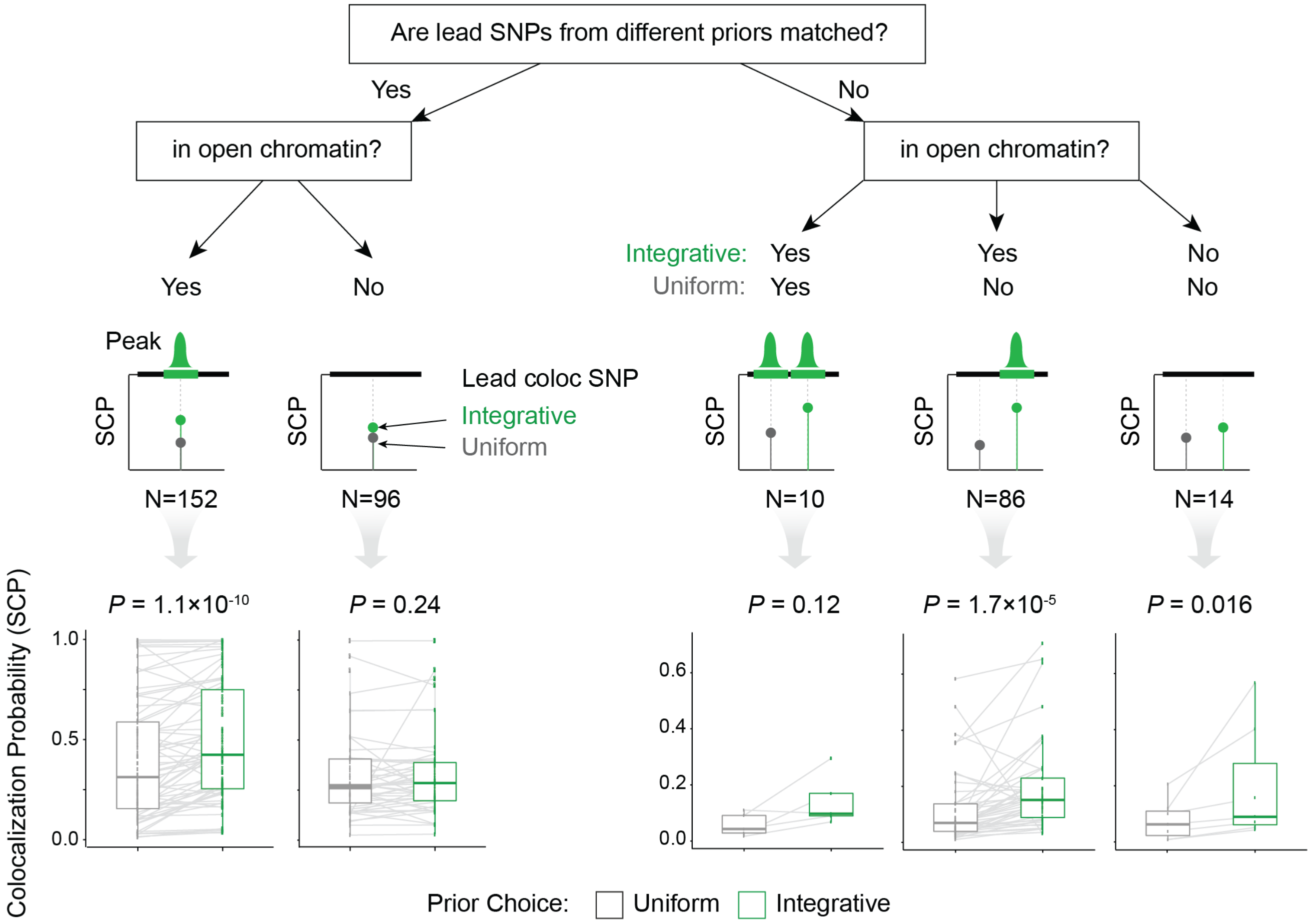

Supplementary Figure 8. SNP colocalization probability of lead SNP for each colocalized cluster. Lead SNP defined as SNP with highest colocalization probability (SCP). Association tests are stratified by open chromatin status and the prior used to discover the gene-SNP pair, and the number of SNPs in each category are noted. All four analyses (GLOM-eGFR, GLOM-UACR, TUBE-eGFR, TUBE-UACR) are combined for comparisons. To compare the increase/decrease in colocalization probability with respect to the prior choice, lead SNPs where the colocalized eSNPs share the same eGene are paired. To test the statistical significance a two-sided paired-sample Wilcoxon test was used. Notably, there are no SNP pairs in which the lead SNP from integrative prior is in closed chromatin and the lead SNP from uniform prior is in open chromatin. The box plots contain the 25th to 75th quartile with median indicated by the middle bar. Lines extend 1.5 times the interquartile range.

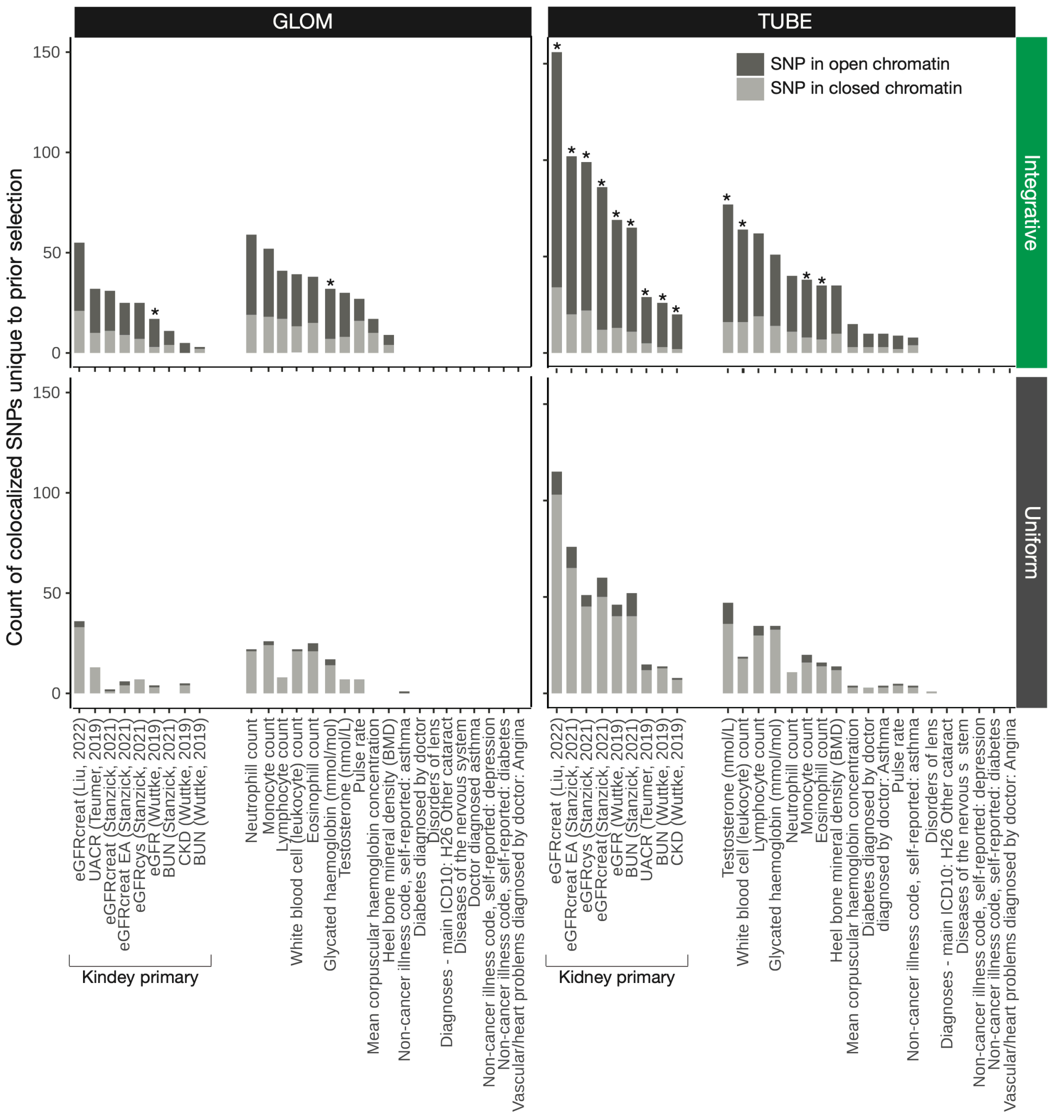

Supplementary Figure 9. Enrichment of colocalized SNPs in open chromatin across primary kidney phenotypes and UK-biobank phenotypes. UK-biobank traits are not significantly genetically correlated with eGFR/UACR ($|r_g| < 0.1$, $P > 0.1$) across different tissues and prior selection. For each colocalized cluster within each GWAS-eQTL-prior pairing, we selected the most probable SNP and tested overlap with kidney open chromatin (see **Methods**). The height of each bar represents the number of colocalized SNPs unique to the results given the specified prior (i.e., only found with uniform prior or only found with integrative prior), with dark grey indicating the proportion of SNPs that overlap open chromatin. A null distribution was generated for each GWAS-tissue-prior pairing using random SNPs matched for eQTL ranking. The enrichment significance was tested with a one-sided Binomial test. *$P < 0.05$. Exact P-values are in Supplementary Data 8.

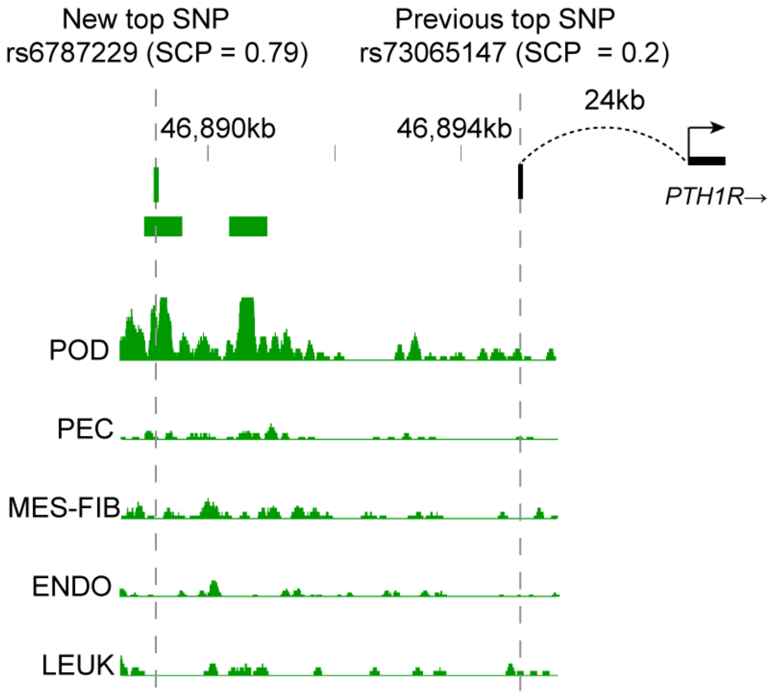

Supplementary Figure 10. Refined top colocalized SNP for GLOM *PTH1R* and UACR. Previously, Teumer et al. identified rs73065147 (black) as the lead SNP in colocalization analysis using array-based GLOM eQTLs (Gillies et at.). Utilizing the integrative prior method identified with high confidence (SCP = 0.79) rs7687229 as the lead *PTH1R* SNP colocalized with UACR (SCP = 0.79), which overlaps an open chromatin peak in podocytes. Vertical dashed lines connect the lead SNPs to their genomic coordinate with an open-chromatin perspective. Horizontal and vertical bar plots depict the genomic range of open chromatin peaks and the pile-up of snATAC-seq reads on the relevant cell types. *PTH1R* parathyroid hormone 1 receptor, POD podocyte, PEC parietal epithelial cell, MES-FIB mesangial-fibroblast, ENDO endothelial, LEUK leukocyte, SCP SNP-level colocalization probability, SNP single nucleotide polymorphism.

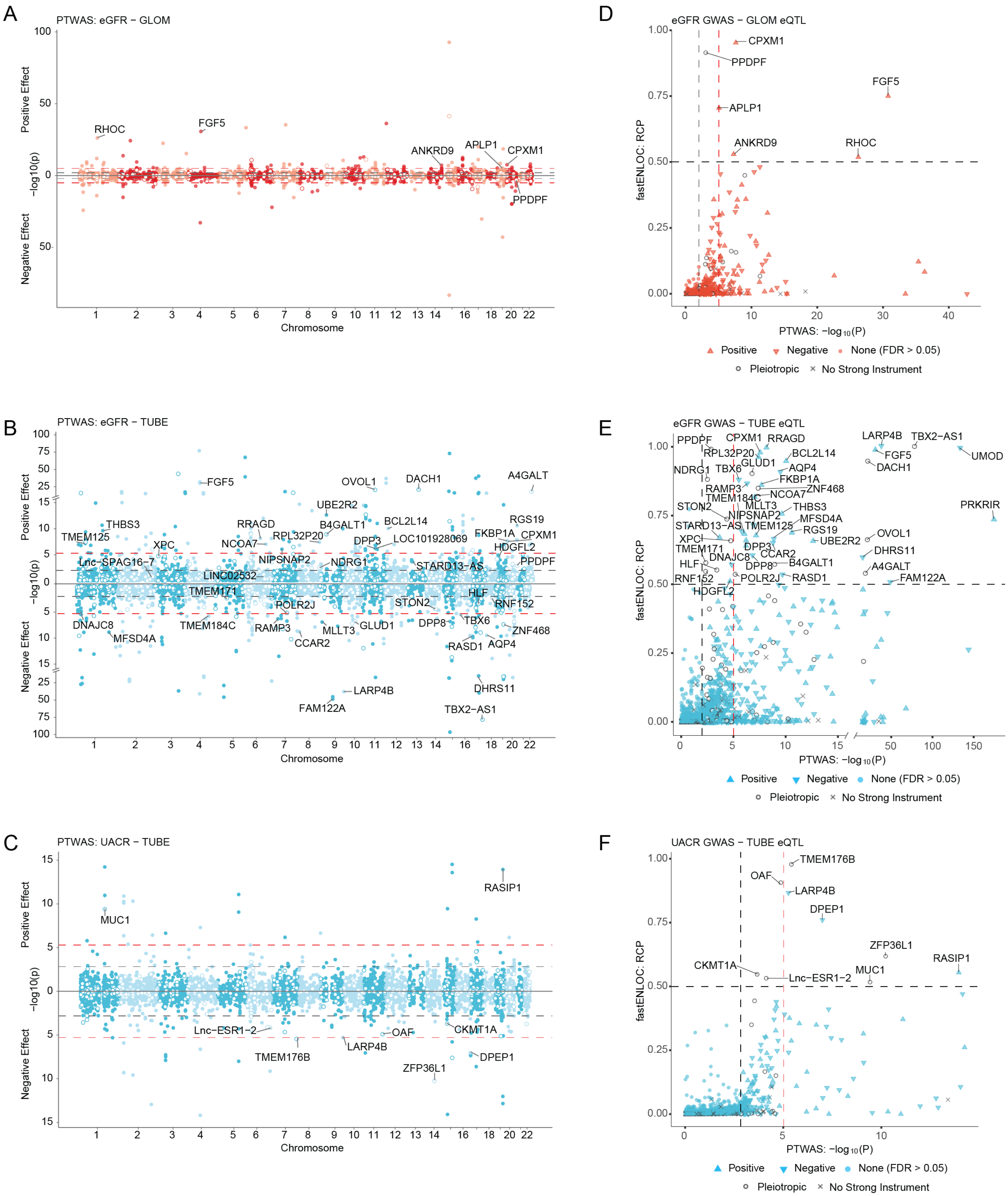
A
PTWAS: eGFR − GLOM
Positive Effect
Negative Effect
−log10(p)
Chromosome
RHOC
FGF5
ANKRD9
APLP1
CPXM1
PPDPF
B
PTWAS: eGFR − TUBE
FGF5
OVOL1
DACH1
A4GALT
UBE2R2
THBS3
TMEM125
RRAGD
B4GALT1
BCL2L14
RGS19
RPL32P20
DPP3
LOC101928069
FKBP1A
CPXM1
XPC
NCOA7
HDGFL2
Lnc−SPAG16−7
NIPSNAP2
NDRG1
STARD13−AS
PPDPF
LINC02532
TMEM171
HLF
POLR2J
STON2
RNF152
DNAJC8
TMEM184C
DPP8
TBX6
MFSD4A
RAMP3
MLLT3
GLUD1
ZNF468
CCAR2
RASD1
AQP4
LARP4B
DHRS11
FAM122A
TBX2−AS1
C
PTWAS: UACR − TUBE
MUC1
RASIP1
Lnc−ESR1−2
OAF
CKMT1A
TMEM176B
LARP4B
DPEP1
ZFP36L1
D
eGFR GWAS − GLOM eQTL
fastENLOC: RCP
PTWAS: −log10(P)
CPXM1
PPDPF
FGF5
APLP1
ANKRD9
RHOC
Positive
Negative
None (FDR > 0.05)
Pleiotropic
No Strong Instrument
E
eGFR GWAS − TUBE eQTL
PPDPF
CPXM1
RRAGD
LARP4B
TBX2−AS1
UMOD
RPL32P20
BCL2L14
FGF5
GLUD1
DACH1
NDRG1
TBX6
AQP4
FKBP1A
RAMP3
ZNF468
TMEM184C
NCOA7
PRKRIR
STON2
MLLT3
THBS3
NIPSNAP2
MFSD4A
STARD13−AS
TMEM125
RGS19
XPC
UBE2R2
OVOL1
TMEM171
DPP3
DHRS11
DNAJC8
CCAR2
HLF
B4GALT1
DPP8
A4GALT
RNF152
POLR2J
RASD1
FAM122A
HDGFL2
F
UACR GWAS − TUBE eQTL
TMEM176B
OAF
LARP4B
DPEP1
ZFP36L1
RASIP1
CKMT1A
MUC1
Lnc−ESR1−2

Supplementary Figure 11. PTWAS Miami plots for **A** eGFR - GLOM, **B** eGFR - TUBE, and **C** UACR - TUBE. Each dot represents a gene, and the genes potentially confounded by pleiotropic effects are indicated with an open circle. Genes with no strong instrument are excluded. PTWAS $-\log_{10}(P)$-values represent the result of a generalized burden test for each gene. Dashed lines indicate thresholds using two multiple testing correction methods, *q*-value ($q \leq 0.05$; light gray) and Bonferroni ($P \leq 9.52\times10^{-6}$; red). Genes with regional colocalization probability (RCP) ≥ 0.5 from the corresponding colocalization analysis are labeled. **D-F** Scatter plots of RCP of top colocalized clusters from fastENLOC and corresponding PTWAS associations for each eGene. PTWAS $-\log_{10}(P)$-values represent the result of a generalized burden test for each gene. The shape of points depicts the type of effect of the PTWAS association, as shown in the figure. Genes with no strong instrument are excluded.

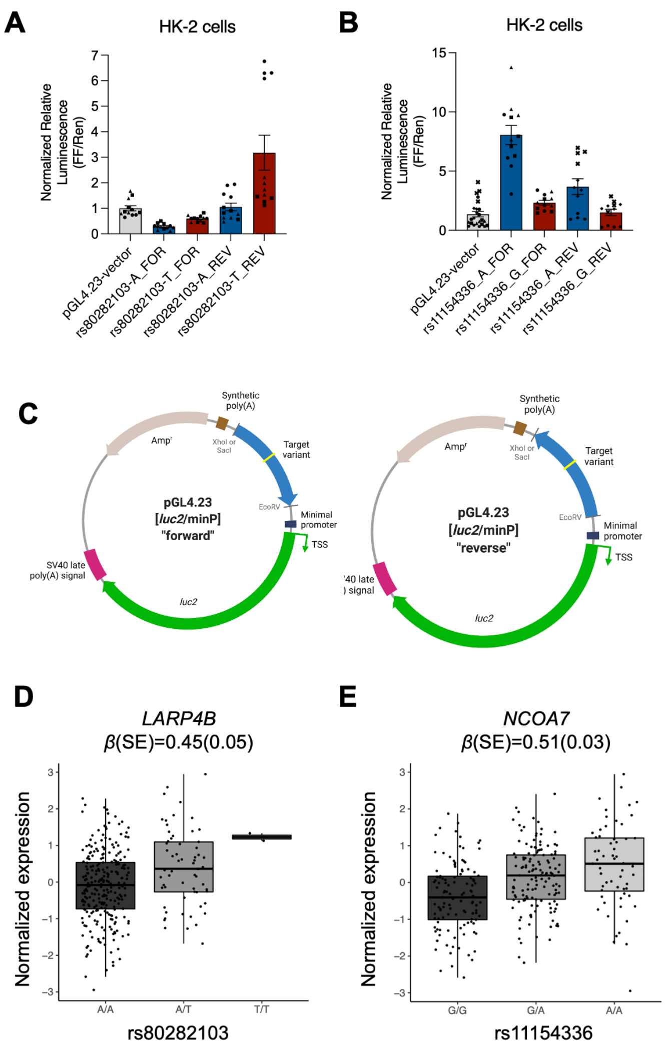

Supplementary Figure 12. A-B Reporter assay in HK-2 cells testing the allele-specific enhancer activity (A) rs80282103 and (**B**) rs11154336. Results are given as ratios of firefly to *Renilla* luciferase activity. Results shown from three independent experiments with quadruplicate measurements in each repeat. *P* from a linear regression model with log-transformed relative luminescence adjusting for batch and orientation = rs80282103: 5.99×10^{-9}, rs11154336: 1.30×10^{-16}. Error bars denote standard error of the mean (SEM). Point shapes denote different batches. (C) Diagrams for the luciferase constructs. **D-E** TUBE eQTL (N=311) boxplots of (D) *LARP4B*-rs80282103 and (E) *NCOA7*-rs11154336. The box plots contain the 25th to 75th quartile with median indicated by the middle bar. Lines extend 1.5 times the interquartile range.

NephQTL2 Tutorial

Welcome to NephQTL2.org!

- From the home page, you can choose to search by gene or variant in either glom or tube.
  - 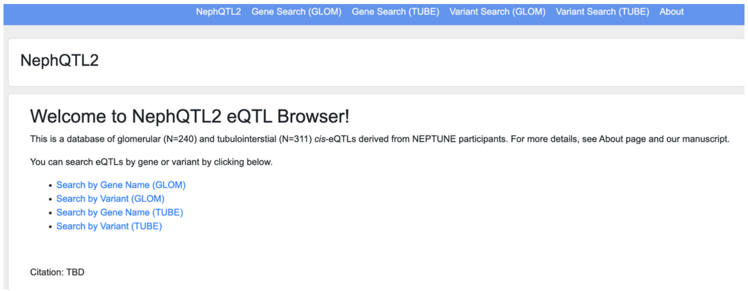

NephQTL2 Gene Search (GLOM) Gene Search (TUBE) Variant Search (GLOM) Variant Search (TUBE) About

NephQTL2

Welcome to NephQTL2 eQTL Browser!

This is a database of glomerular (N=240) and tubulointerstial (N=311) *cis*-eQTLs derived from NEPTUNE participants. For more details, see About page and our manuscript.

You can search eQTLs by gene or variant by clicking below.

- Search by Gene Name (GLOM)
- Search by Variant (GLOM)
- Search by Gene Name (TUBE)
- Search by Variant (TUBE)

Citation: TBD

- Let's start by searching for a gene in the GLOM eQTL
  - Click "Search by Gene Name (GLOM)

Gene Search

- When searching for a gene, you can enter the ensemble ID or the gene symbol. Entering an incomplete symbol will result in all genes that begin with the entry. For example, if you type in 'NPHS', you'll get results for *NPHS1* and *NPHS2*.
  - 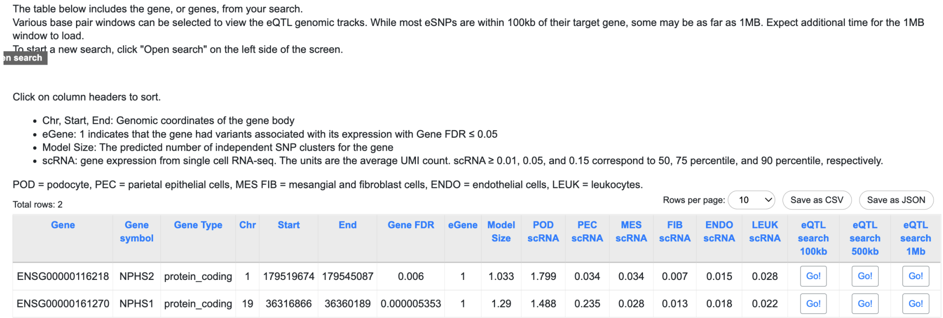

The table below includes the gene, or genes, from your search.
Various base pair windows can be selected to view the eQTL genomic tracks. While most eSNPs are within 100kb of their target gene, some may be as far as 1MB. Expect additional time for the 1MB window to load.
To start a new search, click "Open search" on the left side of the screen.

n search

Click on column headers to sort.

- Chr, Start, End: Genomic coordinates of the gene body
- eGene: 1 indicates that the gene had variants associated with its expression with Gene FDR ≤ 0.05
- Model Size: The predicted number of independent SNP clusters for the gene
- scRNA: gene expression from single cell RNA-seq. The units are the average UMI count. scRNA ≥ 0.01, 0.05, and 0.15 correspond to 50, 75 percentile, and 90 percentile, respectively.

POD = podocyte, PEC = parietal epithelial cells, MES FIB = mesangial and fibroblast cells, ENDO = endothelial cells, LEUK = leukocytes.

Total rows: 2 Rows per page: 10 Save as CSV Save as JSON

Gene	Gene symbol	Gene Type	Chr	Start	End	Gene FDR	eGene	Model Size	POD scRNA	PEC scRNA	MES scRNA	FIB scRNA	ENDO scRNA	LEUK scRNA	eQTL search 100kb	eQTL search 500kb	eQTL search 1Mb
ENSG00000116218	NPHS2	protein_coding	1	179519674	179545087	0.006	1	1.033	1.799	0.034	0.034	0.007	0.015	0.028	Go!	Go!	Go!
ENSG00000161270	NPHS1	protein_coding	19	36316866	36360189	0.000005353	1	1.29	1.488	0.235	0.028	0.013	0.018	0.022	Go!	Go!	Go!

- The results table will show the gene, or genes, along with additional gene level information, including the type of gene and genomic position.
- Genes with FDR < 0.05 are considered "eGenes", which are genes with at least one variant associated with its expression.
- The model size indicates the number of independent eQTL clusters associated with the gene, it is a continuous variable, so here we can conclude each gene has 1 confident eQTL cluster.
- We've also included the average UMI count from scRNA data. Here values greater than 0.15 are within the 90th percentile of expressed genes, so as expected, *NPHS1* and *NPHS2* are both highly expressed in the podocyte.
- We include three different options for viewing the eQTL results, each with varying flanking regions form the gene body. Note that while some eSNPs are within 100kb, we tested all SNPs within a Mb. If you decided to view the Mb region, note that it will take longer to load results.

- Before we explore the results, let's take a step back and see how to search by variant.

  - Click "Variant Search (GLOM)" at the top of the page

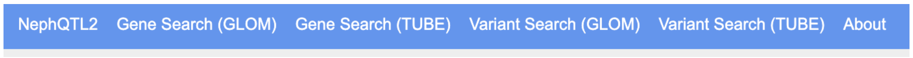

<u>Variant Search</u>

- Let's search for "rs28373331"
- The results will include associations for every glomerular expressed gene within 1Mb from the SNP. For each gene, we have the same information as the gene search page, but now we also have SNP information, including the Beta (Effect size/direction) and the unadjusted nominal p-value from single-SNP association analysis with MatrixEQTL.
- You can sort by gene FDR to find the most significantly associated eGene, which here is *SBSPON*.

Gene	Gene symbol	Gene Type	Gene-level FDR	eGene	Model Size	SNP Beta	SNP P-Value	POD scRNA	PEC scRNA	MES scRNA	FIB scRNA	ENDO scRNA	LEUK scRNA	eQTL search 100kb	eQTL search 500kb	eQTL search 1Mb
ENSG00000164764	SBSPON	protein_coding	3.015e-54	1	1.029	0.809	3.148e-48	0.389	0.065	0.138	0.075	0.006	0.017	Go!	Go!	Go!
ENSG00000253636	.	lncRNA	2.958e-36	1	1.273	0.885	5.678e-33	.	.	.	.	.	.	Go!	Go!	Go!
ENSG00000104343	UBE2W	protein_coding	0.183	0	0.382	0.066	0.447	.	.	.	.	.	.	Go!	Go!	Go!
ENSG00000175606	TMEM70	protein_coding	0.228	0	0.333	0.088	0.187	0.015	0.007	0.006	0	0.008	0	Go!	Go!	Go!
ENSG00000121039	RDH10	protein_coding	0.368	0	0.223	0.072	0.262	0.055	0.064	0.015	0.036	0.027	0	Go!	Go!	Go!
ENSG00000272254	.	lncRNA	0.395	0	0.209	0.268	0.004	.	.	.	.	.	.	Go!	Go!	Go!
ENSG00000235531	MSC-AS1	lncRNA	0.425	0	0.192	-0.085	0.35	0.012	0.605	0.111	0.117	0.023	0.044	Go!	Go!	Go!
ENSG00000147601	TERF1	protein_coding	0.427	0	0.192	0.106	0.114	0.092	0.1	0.11	0.085	0.051	0.039	Go!	Go!	Go!
ENSG00000154582	ELOC	protein_coding	0.47	0	0.169	-0.043	0.522	0.05	0.039	0.034	0.043	0.042	0	Go!	Go!	Go!

- From this table, we can learn that *SBSPON* is a protein coding gene with highest expression in the podocyte. Let's click on 'Go!' for the 100kb eQTL search.
- This brings us to the eQTL results page.

eQTL Results Page

- The Manhattan plot at the top displays results from the single-SNP analysis with MatrixEQTL, where the x-axis is the genomic position, and the y-axis is the significance. Each point is a variant with the coloring reflecting linkage disequilibrium, or LD, with the lead SNP, indicated by a diamond.

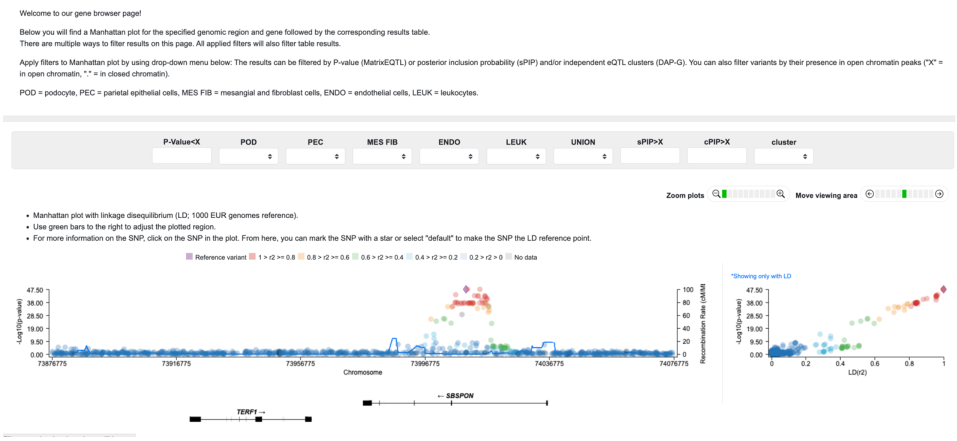

- Below the Manhattan plot is a gene track, however results on the Manhattan plot are only for the selected gene, *SBSPON*. To explore other genes in the region, you need to start from the Gene Search page again.

- You can use zoom and scroll the plot using these bars, let's zoom in on the signal

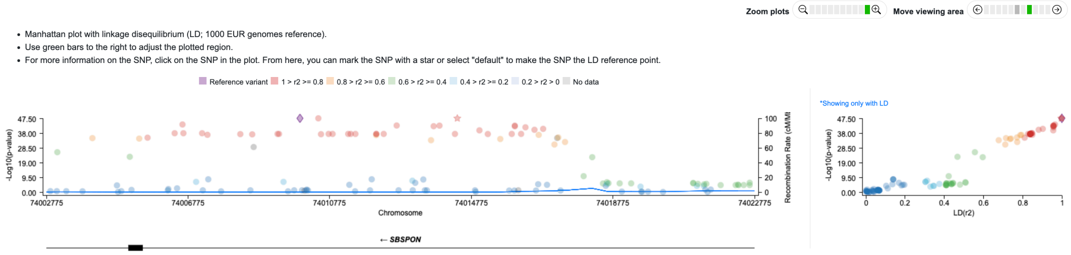

- You can hover over the variant to see the beta and p-value, mark it as a favorite by clicking the star, or set it as the LD reference SNP. Let's 'star' the top three variants.

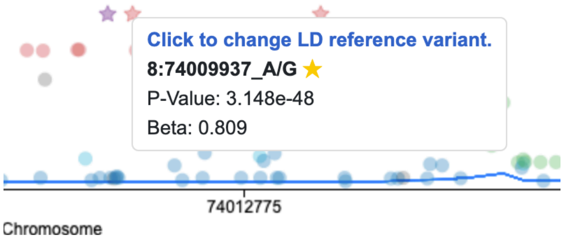

- You can also use the dropdown bars on the top of the screen to filter the plot

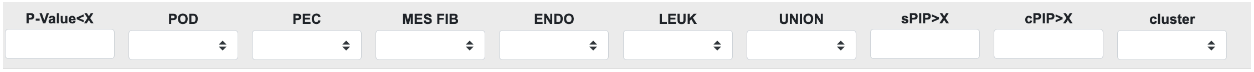

  - For example, if we chose "X" under "POD", we would see all variants that are in podocyte open chromatin, which includes one of the top SNPs. We can also filter for the union of all cell type peaks with the "UNION" filter option.

Variant Annotations

- We can explore fine-mapped variants by clicking "Filter associated variants by credible sets". Here we can see that there are three independent clusters of SNPs associated with *SBSPON* expression, let's check out the first one.

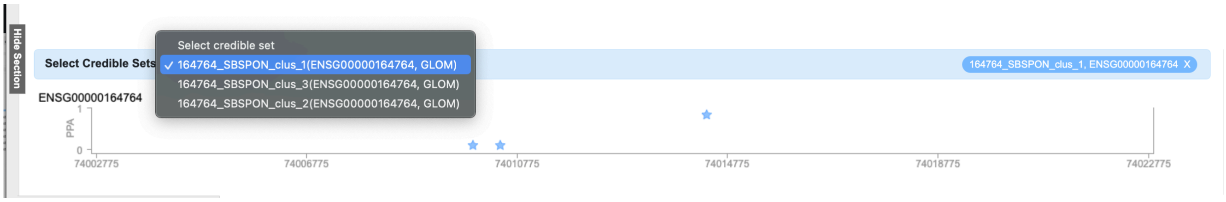

- We can see that this fine-mapped cluster contains all three of the most significant SNPs that we marked with stars, with one variant having higher posterior probability than the other two.

- You can hover over each SNP to get more information

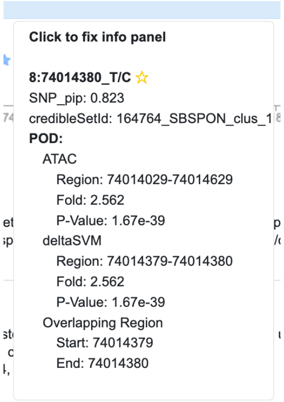

- In addition to the plots, the filtered results are also shown in the table at the bottom of the page, which we will discuss shortly.

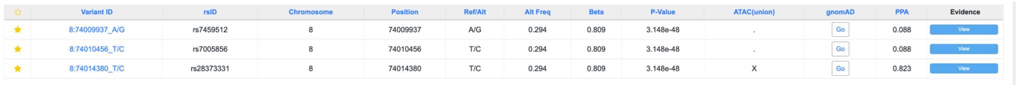

☆	Variant ID	rsID	Chromosome	Position	Ref/Alt	Alt Freq	Beta	P-Value	ATAC(union)	gnomAD	PPA	Evidence
★	8:74009937_A/G	rs7459512	8	74009937	A/G	0.294	0.809	3.148e-48	.	Go	0.088	View
★	8:74010456_T/C	rs7005856	8	74010456	T/C	0.294	0.809	3.148e-48	.	Go	0.088	View
★	8:74014380_T/C	rs28373331	8	74014380	T/C	0.294	0.809	3.148e-48	X	Go	0.823	View

- We can now look at the epigenomic annotations associated with these variants. Let's start with ATACseq tracks

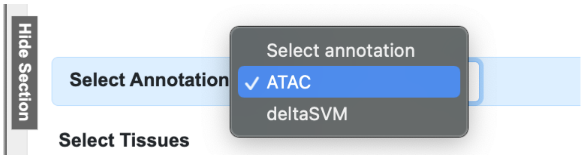

- We can click the cell type of interest to highlight the corresponding tracks. We can click on deltaSVM to add the tracks as well.

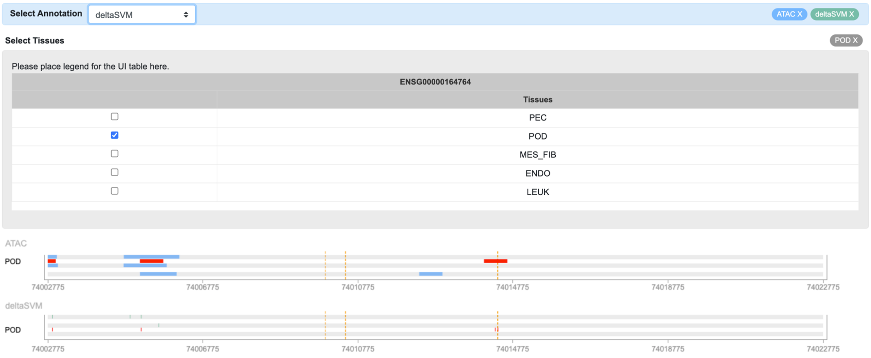

- Notice that the starred variants are highlighted here with an orange dashed line. We can see that the variant with the highest posterior probability falls within podocyte-specific open chromatin and is deltaSVM positive, and thus predicted to have a regulatory effect.
- Selecting the open chromatin and/or deltaSVM tracks also filters the results table below. So here we have one variant that has satisfied all filters.

Total rows: 1 — Compare annotated regions: Or — Rows per page: 10 — Save as CSV — Save as JSON

☆	Variant ID	rsID	Chromosome	Position	Ref/Alt	Alt Freq	Beta	P-Value	ATAC(union)	gnomAD	PPA	Annotation Overlap	POD	Evidence
★	8:74014380_T/C	rs28373331	8	74014380	T/C	0.294	0.809	3.148e-48	X	Go	0.823	74014029-74014629	ATAC: 74014029-74014629, deltaSVM: 74014379-74014380	View

- Let's remove all the filters we applied and view the full table of results

Results Table

- Here we can see the genomic position of the SNP, the ref/alt allele, the alt allele frequency and the beta and p-value from single-SNP eQTL analysis with MatrixEQTL.
- We can click on the headers of the columns to sort.
- We can see our top starred SNPs with indistinguishable effects, with one SNP falling in an open chromatin peak

Total rows: 881 — Compare annotated regions: Or — Rows per page: 10 — Save as CSV — Save as JSON

☆	Variant ID	rsID	Chromosome	Position	Ref/Alt	Alt Freq	Beta	P-Value	ATAC(union)	gnomAD	Evidence
★	8:74009937_A/G	rs7459512	8	74009937	A/G	0.294	0.809	3.148e-48	.	Go	View
★	8:74010456_T/C	rs7005856	8	74010456	T/C	0.294	0.809	3.148e-48	.	Go	View
★	8:74014380_T/C	rs28373331	8	74014380	T/C	0.294	0.809	3.148e-48	X	Go	View

- We can click on the "Evidence" tab to view ATAC and deltaSVM annotations. Note that higher deltaSVM scores indicate higher predicted functionality, with a deltaSVM > 2.0 corresponding to the 91st percentile. So, the deltaSVM score of 2.94 in the podocyte indicates the SNP has a high

probability of regulating gene expression in the podocyte.

☆	Variant ID	rsID	Chromosome	Position	Ref/Alt	Alt Freq	Beta	P-Value	ATAC(union)	gnomAD	Evidence
★	8:74009937_A/G	rs7459512	8	74009937	A/G	0.294	0.809	3.148e-48	.	Go	View
★	8:74010456_T/C	rs7005856	8	74010456	T/C	0.294	0.809	3.148e-48	.	Go	View
★	8:74014380_T/C	rs28373331	8	74014380	T/C	0.294	0.809	3.148e-48	X	Go	View

ATAC	POD ATAC	PEC ATAC	MES FIB ATAC	ENDO ATAC	LEUK ATAC
	X	.	.	.	.

dSVM	POD dSVM	PEC dSVM	MES FIB dSVM	ENDO dSVM	LEUK dSVM
	2.94	.	.	.	.

DAP-G	SNP PIP	cluster PIP	cluster	cluster R2
	0.823	1	1	1

- Under the evidence tab, we can also see fine-mapping results, including SNP posterior probability, eQTL cluster assignment, cluster probability and R2 (correlation of SNPs within the cluster).

- If you're interested in exploring other genes associated with a SNP, you can click on the SNP to bring you to the variant search results page.

This concludes our tutorial. Please contact us with any questions or inquiries and be sure to check out our manuscript linked on the main page.

Members of the Nephrotic Syndrome Study Network (NEPTUNE)

NEPTUNE Enrolling Centers
Atrium Health Levine Children's Hospital, Charlotte, SC: S Massengill*
Cleveland Clinic, Cleveland, OH: K Dell*, J Sedor**, B Martin#
Children's Hospital, Los Angeles, CA: K Lemley*, S Sharma#
Children's Mercy Hospital, Kansas City, MO: T Srivastava*, K Markus#
Cohen Children's Hospital, New Hyde Park, NY: C Sethna*, S Vento #
Columbia University, New York, NY: P Canetta*, A Pradhan#
Emory University, Atlanta, GA: L Greenbaum*, CS Wang**, E Yun#
Harbor-University of California Los Angeles Medical Center: S Adler*, J LaPage#
Johns Hopkins Medicine, Baltimore, MD: M Atkinson*, M Williams#
Kansas University, Kansas City, KS: E McCarthy*
Mayo Clinic, Rochester, MN: F Fervenza*, M Hogan**, J Lieske*#
Medical University of South Carolina, D Selewski*, C Conley#
Montefiore Medical Center, Bronx, NY: F Kaskel*, M Ross*, P Flynn#
NIDDK Intramural, Bethesda MD: J Kopp*
New York University Medical Center, New York, NY: L Malaga-Dieguez*, O Zhdanova**, B Pace#
The Ohio State University College of Medicine, Columbus, OH: S Almaani*
Stanford University, Stanford, CA: R Lafayette*, S Dave#
Temple University, Philadelphia, PA: I Lee*, S Quinn-Boyle#
Texas Children's Hospital at Baylor College of Medicine, Houston, TX: S Shah*
University Health Network Toronto: H Reich *, M Hladunewich**, P Ling#, M Romano#
University of California at San Francisco, San Francisco, CA: P Brakeman*
University of Colorado Anschutz Medical Campus, Aurora, CO: A Podoll*
University of Miami, Miami, FL: A Fornoni*, C Bidot#
University of Michigan, Ann Arbor, MI: M Kretzler*, D Gipson*, A Williams#, C Klida#
University of North Carolina, Chapel Hill, NC: V Derebail*, K Gibson*, A Froment#, F Ochoa-Toro#
University of Pennsylvania, Philadelphia, PA: L Holzman*, K Meyers**, K Kallem#, A Swenson#
University of Texas San Antonio, San Antonio, TX: K Sharma*
University of Texas Southwestern, Dallas, TX: K Sambandam*, Z Wang#, M Rogers#
University of Washington, Seattle, WA: A Jefferson*, S Hingorani**, K Tuttle**§, L Manahan #, E Pao#, K Kuykendall K§
Wake Forest University Baptist Health, Winston-Salem, NC: JJ Lin*, Stefanie Baker#
Washington University in St. Louis, St. Louis, MO: V Dharnidharka*

Data Analysis and Coordinating Center: M Kretzler*, L Barisoni**, C Gadegbeku**, B Gillespie**, D Gipson**, L Holzman**, L Mariani**, M Sampson**, J Sedor**, A Smith**, J Zee**, G Alter, H Desmond, S Eddy, D Fermin, M Larkina, S Li, S Li, CC Lienczewski, T Mainieri, R Scherr, A Williams.

Digital Pathology Committee: Carmen Avila-Casado (University Health Network, Toronto), Serena Bagnasco (Johns Hopkins University), Clarissa Cassol (Arakana), Lihong Bu (Mayo Clinic), Shelley Caltharp (Emory University), Dawit Demeke (University of Michigan), Brenda Gillespie (University of Michigan), Jared Hassler (Temple University), Leal Herlitz (Cleveland Clinic), Stephen Hewitt (National Cancer Institute), Jeff Hodgin (University of Michigan), Danni Holanda (Arkana), Neeraja Kambham (Stanford University), Kevin Lemley (Children's Hospital of Los Angeles), Laura Mariani (University of Michigan), Nidia Messias (Washington University), Alexei Mikhailov (Wake Forest), Behzad Najafian (University of Washington), Matthew Palmer (University of Pennsylvania), Avi Rosenberg (Johns Hopkins University), Virginie Royal (University of Montreal), Barry Stokes (Columbia University), David Thomas (Duke University), Michifumi Yamashita (Cedar Sinai), Hong Yin (Emory University) Jarcy Zee (University of Pennsylvania), Yiqin Zuo (University of Miami), Co-Chairs: Laura Barisoni (Duke University) and Cynthia Nast (Cedar Sinai).

*Principal Investigator; **Co-investigator; #Study Coordinator
§Providence Medical Research Center, Spokane, WA
