# Supplementary material for: Mapping genomic regulation of kidney disease and traits through high-resolution and interpretable eQTLs
Source: Nat Commun. 2023 Apr 19;14:2229. doi: 10.1038/s41467-023-37691-7 (PMC10115815; doi:10.1038/s41467-023-37691-7)
Supplement: Supplementary file 2 — Description of Additional Supplementary Files [file 41467_2023_37691_MOESM2_ESM.pdf]

Description of Additional Supplementary Files

File Name: Supplementary Data 1
Description: **NEPTUNE cohort summary.** This table shows the demographic and clinical characteristics of NEPTUNE participants included in eQTL analyses. FSGS= focal segmental glomerular sclerosis, MCD = minimal change disease, MN = membranous nephropathy. Continuous variables are presented as mean (standard deviation) and discrete variables are count (percentage).

File Name: Supplementary Data 2
Description: **Enrichment of UACR or eGFR heritability stratified by cell-type grouping.** This table shows results of S-LDSC with peaks residing in cell-group-specific peaks (C1 - C4). See figure 2C. SD and P-values of heritability enrichment were calculated from the block jackknife method implemented in the LDSC software. h2 = heritability, SE= standard error.

File Name: Supplementary Data 3
Description: **Enrichment of eSNPs in optimized peaks.** This table shows enrichment estimates for optimized peaks using TORUS. CRE = cis-regulatory element, LogOR = logarithm of the odds ratio, SE = standard error of logOR, ENDO = endothelial, LEUK = leukocytes, MES-FIB = mesangial/fibroblast, PEC = parietal epithelial cells, POD = podocyte.

File Name: Supplementary Data 4
Description: **eGenes from glomerular (GLOM) and tubulointerstitial (TUBE) compartments.** This table shows gene-level eQTL results for GLOM and TUBE. Genes not expressed in a tissue compartment will be missing. eGene = gene-level FDR < 0.05, Model Size = number of independent associations (continuous variable).

File Name: Supplementary Data 5
Description: **Top SBSPON associations from single-SNP association study with MatrixEQTL.** This table shows indistinguishable association strength prior to Bayesian fine-mapping. Beta = genotype effect on gene expression, P-value= nominal unadjusted p-value is the result of linear regression model comparing expression to genotype.

File Name: Supplementary Data 6
Description: **Results of S-LDSC analysis using the GWAS of kidney primary phenotypes and UK-biobank phenotypes not significantly genetically correlated with eGFR/UACR.** This table shows the raw data of Figure 4B and Supplementary figures S5B. SD and P-values of heritability enrichment were calculated from the block jackknife method implemented in the LDSC software. h2 = heritability, SE= standard error.

File Name: Supplementary Data 7
Description: **Significant colocalization results.** This table shows the colocalization results for all loci with RCP >= 0.5 identified by fastEnloc. All tissue-trait pairs are included (GWAS included: eGFRcreat (Liu et al., 2022), eGFRcys (Stanzick et al., 2021), eGFR (Wuttke et al, 2019), UACR (Tuemer et al., 2019)). For each gene, the lead SNP (highest posterior probability) is included. For

loci with indistinguishable lead SNPs, multiple SNPs are included. RCP = regional colocalization probability, SCP = SNP colocalization probability.

File Name: Supplementary Data 8
Description: **Enrichment of colocalized GWAS variants in open chromatin.** P-value from a one-sided ('greater') Binomial test of the observed overlap using the estimated expectations.

File Name: Supplementary Data 9
Description: **Significant PTWAS associations.** This table shows the PTWAS results for all loci with q-value (FDR) < 0.05. All tissue-trait pairs are included (GLOM/UACR, GLOM/eGFR, TUBE/UACR, TUBE/eGFR). P-value = Significance from GAMBIT gene-based test chi-square statistic (two-sided). Unadjusted for multiple testing. No covariates used. PTWAS = probabilistic transcriptome-wide association study, Number of SNPs = number of SNPs used for analysis, Cumulative PIP = cumulative eQTL posterior probabilities for SNPs used in analysis, Number of Instruments = Number of eligible instruments (signal clusters with a SNP exceeding SNP PIP threshold of 0.5, if no SNP PIP > 0.5, Number of Instruments == "No strong instrument"), Estimated effect size = positive indicates increase in gene expression is associated with an increase in the trait (UACR or eGFR), I^2 = statistic to assess the heterogeneity of estimated effects across eligible signal clusters (> 0.1 used to identify potential pleiotropy).

File Name: Supplementary Data 10
Description: **RNAi in Drosophila nephrocytes.** This table shows results from RNAi knock-down of selected genes in the *Drosophila* nephrocytes. Nephrocyte expression rank = ranked expression from 1 to 15,000 with 1 being highest expressed, DIOPT= conservation score, ANF-RFP assay = measures secretion (atrium natriuretic factor - red fluorescent protein), Dextran assay = measures reabsorption. P-value = two-sided t-test comparing assay to control. Note: p-value of Rho1 is <0.001, but it is because Rho1 has higher pMAR uptake than control. The RNAi target sequences could be found at Flybase with Bloomington Stock Center RNAi line #.

File Name: Supplementary Data 11
Description: **List of primers used to generate luciferase reporter constructs.** "Plus" primer pairs are used to amplify target genomic sequences and generate "forward orientation" constructs; "Minus" primer pairs are used to generate "reverse orientation" constructs. "Q5SDM" primer pairs were generated using NEBaseChanger (v1.3.3) for use with Q5 Site-Directed Mutagenesis Kit (NEB, #E0554S). Base substitutions to generate alternate alleles are indicated in bold/underline.

File Name: Supplementary Data 12
Description: **Resource List**
