# Supplementary material for: Mapping genomic regulation of kidney disease and traits through high-resolution and interpretable eQTLs
Source: Nat Commun. 2023 Apr 19;14:2229. doi: 10.1038/s41467-023-37691-7 (PMC10115815; doi:10.1038/s41467-023-37691-7)
Supplement: Supplementary file 4 — Reporting Summary [file 41467_2023_37691_MOESM4_ESM.pdf]

nature portfolio

Corresponding author(s): Matthew G. Sampson

Last updated by author(s): 03/06/2023

Reporting Summary

Nature Portfolio wishes to improve the reproducibility of the work that we publish. This form provides structure for consistency and transparency in reporting. For further information on Nature Portfolio policies, see our Editorial Policies and the Editorial Policy Checklist.

Statistics

For all statistical analyses, confirm that the following items are present in the figure legend, table legend, main text, or Methods section.

n/a	Confirmed	
☐	☒	The exact sample size (*n*) for each experimental group/condition, given as a discrete number and unit of measurement
☐	☒	A statement on whether measurements were taken from distinct samples or whether the same sample was measured repeatedly
☐	☒	The statistical test(s) used AND whether they are one- or two-sided<br>*Only common tests should be described solely by name; describe more complex techniques in the Methods section.*
☐	☒	A description of all covariates tested
☐	☒	A description of any assumptions or corrections, such as tests of normality and adjustment for multiple comparisons
☐	☒	A full description of the statistical parameters including central tendency (e.g. means) or other basic estimates (e.g. regression coefficient) AND variation (e.g. standard deviation) or associated estimates of uncertainty (e.g. confidence intervals)
☐	☒	For null hypothesis testing, the test statistic (e.g. *F*, *t*, *r*) with confidence intervals, effect sizes, degrees of freedom and *P* value noted<br>*Give P values as exact values whenever suitable.*
☐	☒	For Bayesian analysis, information on the choice of priors and Markov chain Monte Carlo settings
☐	☒	For hierarchical and complex designs, identification of the appropriate level for tests and full reporting of outcomes
☐	☒	Estimates of effect sizes (e.g. Cohen's *d*, Pearson's *r*), indicating how they were calculated

Our web collection on statistics for biologists contains articles on many of the points above.

Software and code

Policy information about availability of computer code

Data collection

No software was used.

Data analysis

Cell Ranger ATAC (v1.1.0)
SnapATAC (v1; 2019-09-19)
MACS2 (v2.2.7.1)
gkmQC (v1.0)
Harmony (v0.1)
were used to process snATAC-seq data

Seurat (v3.0.2) was used for processing snRNA-seq data.

fastQC (v0.11.5)
fastQScreen (v0.11.4)
Piccard tools (v2.4.1)
STAR (v2.6.0a)
StringTie (v2.1.4)
edgeR (v3)
were used for processing bulk kidney RNA-seq datasets

GotCloud (v1.12.3)

VCFtools (v0.1)
PLINK (v1.9)
HardyWeinberg R (v3.5.1)
were used for processing whole-genome sequencing datasets

R packages of
MatrixEQTL (v2.3)
PEER (v1.3)
were used for single-SNP eQTL analysis

TORUS
DAP-G
were used for enrichment multi-SNP eQTL fine-mapping analysis.

deltaSVM, gkm-SVM and LS-GKM (v.0.1.1) were used to predict functional regulatory variants.

LDSC (v1.0.1) was used for estimating the proportion and enrichment of heritability.

fastENLOC (v1.0) was used for probabilistic colocalization analysis.

PTWAS (v1.0) and GAMBIT were used for probabilistic TWAS analysis.

R 3.5 and Python 3.7 with 3rd-party package (scipy) were used to perform statistical analysis.

IGV (2.12.3), LocusZoom, ggplot2 were used for visualizing open chromatin, GWAS, eQTL datasets.

For manuscripts utilizing custom algorithms or software that are central to the research but not yet described in published literature, software must be made available to editors and reviewers. We strongly encourage code deposition in a community repository (e.g. GitHub). See the Nature Portfolio guidelines for submitting code & software for further information.

Data

Policy information about availability of data

All manuscripts must include a data availability statement. This statement should provide the following information, where applicable:

- Accession codes, unique identifiers, or web links for publicly available datasets
- A description of any restrictions on data availability
- For clinical datasets or third party data, please ensure that the statement adheres to our policy

Raw data used to generate results are available through NEPTUNE (https://www.neptune-study.org/ancillary-studies). The processed data of kidney fine-mapped eQTL and chromatin accessibility analysis are publicly available online at the NephQTL2 (https://www.nephqtl2.org). We utilized GotCloud with the hg19 resource files available at https://genome.sph.umich.edu/wiki/GotCloud:_Genetic_Reference_and_Resource_Files. Single-cell ATAC-and RNA-seq datasets were downloaded from GEO website (GSE151302). Bulk ATAC-seq data (human kidney samples) is from Dr. Chakravarti's laboratory & Lee et al., 2022 (https://www.biorxiv.org/content/10.1101/2022.04.19.488795v1.abstract). Bulk DNase-seq data from ENCODE (ENCSR543YPH for kidney, ENCSR141VGA for lung, ENCSR148VUP for HMP, ENCSR272RQX for muscle, ENCSR649KBB for brain, ENCFF354YDR for CMP, ENCSR911LTI for heart. Bulk RNAseq and eQTL tissue-specific all SNP gene associations were downloaded from the GTEx consortium (https://storage.googleapis.com/gtex_analysis_v8/rna_seq_data/GTEx_Analysis_2017-06-05_v8_RNASeQCv1.1.9_gene_reads.gct.gz & https://console.cloud.google.com/storage/browser/gtex-resources). Datasets used to compare eQTL effect sizes were downloaded from https://nephqtl.org/, The Susztak Lab (https://susztaklab.com/Kidney_eQTL/download.php), and GTEx (https://console.cloud.google.com/storage/browser/gtex-resources). Summary statistics of eGFR/UACR GWAS were downloaded from CKDGen Consortium (https://ckdgen.imbi.uni-freiburg.de), GWAS catalog (https://www.ebi.ac.uk/gwas/studies/GCST90100220), and UK Biobank (https://pan.ukbb.broadinstitute.org/downloads/index.html & https://docs.google.com/spreadsheets/d/1AeeADtT0U1AukliiNyiVzVRdLYPkTbruQSk38DeutU8/edit#gid=268241601).

Human research participants

Policy information about studies involving human research participants and Sex and Gender in Research.

Reporting on sex and gender	NEPTUNE participants are composed with 205 (61.7%) males and 127 (38.2%) females. snATAC-seq/snRNA-seq data were produced from kidney samples of three males and two females.
Population characteristics	NEPTUNE participants included in eQTL analysis are composed of White/Caucasian (54.5%), Black/African American (25.0%), Asian/Asian American (9.6%), and Other/Unknown/Multi-Racial (10.9%). The detailed information of human participants is provided in Supplementary Table S1. snATAC-seq/snRNA-seq data were produced from kidney samples from five human participants (Age: 50-62; four Non-Hispanic White and one Hispanic).
Recruitment	Samples were recruited into the NEPTUNE study based on their proteinuric kidney disease .Deidentified human kidney samples were obtained from microdissection for the eQTL data. There were no recruitment biases.
Ethics oversight	Institution review board of NEPTUNE study approved the deidentified human kidney samples obtained from microdissection.

Ethics oversight | Research was approved by IRBs at Boston Children's and Washington University.

Note that full information on the approval of the study protocol must also be provided in the manuscript.

Field-specific reporting

Please select the one below that is the best fit for your research. If you are not sure, read the appropriate sections before making your selection.

☒ Life sciences ☐ Behavioural & social sciences ☐ Ecological, evolutionary & environmental sciences

For a reference copy of the document with all sections, see nature.com/documents/nr-reporting-summary-flat.pdf

Life sciences study design

All studies must disclose on these points even when the disclosure is negative.

Sample size	332 human kidney samples with both whole-genome sequencing and RNA-seq data were used in eQTL analysis. Five kidney samples with snRNA/snATAC-seq were used in single-cell analysis. No sample size calculations were performed and sample sizes are the largest possible given available tissue.
Data exclusions	No data were excluded from the analysis.
Replication	The effect size of eQTL signals were correlated with that of publicly available eQTL datasets. Open-chromatin mapping of single-cell chromatin accessibility analysis were compared with that of bulk kidney samples from different individual. Results of colocalization and TWAS were compared in gene level and have inferential reproducibility. For nephrocytes ANF-RFP/Dextran uptake assay, 20 nephrocytes were analyzed from each of 3 flies per indicated genotype. For luciferase report assay, 12 replicates were analyzed per indicated genotype. All replication analyses were successful.
Randomization	No randomization for eQTL, single-cell, probabilistic colocalization/TWAS analysis. Adjusted covariates of eQTL analysis are age, sex, batch, 4 genotype PCs, and PEER factors. A random subset (N=14) of the qualifying genes was selected for functional follow up with nephrocyte assay.
Blinding	Blinding was not relevant to our study because participants were not allocated into experimental groups.

Reporting for specific materials, systems and methods

We require information from authors about some types of materials, experimental systems and methods used in many studies. Here, indicate whether each material, system or method listed is relevant to your study. If you are not sure if a list item applies to your research, read the appropriate section before selecting a response.

Materials & experimental systems

n/a	Involved in the study	
☒	☐	Antibodies
☐	☒	Eukaryotic cell lines
☒	☐	Palaeontology and archaeology
☐	☒	Animals and other organisms
☒	☐	Clinical data
☒	☐	Dual use research of concern

Methods

n/a	Involved in the study	
☒	☐	ChIP-seq
☒	☐	Flow cytometry
☒	☐	MRI-based neuroimaging

Eukaryotic cell lines

Policy information about cell lines and Sex and Gender in Research

Cell line source(s)	HK-2 (human, ATCC CRL-2190)
Authentication	None of cell lines have been authenticated.
Mycoplasma contamination	No indication of contamination
Commonly misidentified lines (See ICLAC register)	No commonly misidentified cell lines

Animals and other research organisms

Policy information about studies involving animals; ARRIVE guidelines recommended for reporting animal research, and Sex and Gender in Research

Laboratory animals	Drosophila melanogaster, young adult fruit flies hatched within three days.
Wild animals	No wild animals were used in this study.
Reporting on sex	Although sex is not expected to affect the results, only female flies were used due to larger size (and easier to dissect).
Field-collected samples	No field collected samples were used in this study.
Ethics oversight	No ethical approve needed. No vertebrate animal models involved.

Note that full information on the approval of the study protocol must also be provided in the manuscript.
